# Blood-glucose regulator design for diabetics based on LQIR-driven Sliding-Mode-Controller with self-adaptive reaching law

**DOI:** 10.1371/journal.pone.0314479

**Published:** 2024-11-27

**Authors:** Omer Saleem, Jamshed Iqbal

**Affiliations:** 1 Department of Electrical Engineering, National University of Computer and Emerging Sciences, Lahore, Pakistan; 2 School of Computer Science, Faculty of Science and Engineering, University of Hull, Hull, United Kingdom; University of Shanghai for Science and Technology, CHINA

## Abstract

Type I Diabetes is an endocrine disorder that prevents the pancreas from regulating the blood glucose (BG) levels in a patient’s body. The ubiquitous Linear-Quadratic-Integral-Regulator (LQIR) is an optimal glycemic regulation strategy; however, it is not resilient enough to withstand measurement noise and meal disruptions. The Sliding-Mode-Controller (SMC) yields robust BG regulation effort at the expense of a discontinuous insulin infusion rate that perturbs the BG concentrations. Hence, the novel contribution of this article is the formulation of a hybridized LQIR-driven SMC strategy that retrieves the benefits of the aforesaid control schemes while avoiding their inherent problems. The proposed control approach is realized by linearly combining a glycemic LQIR law with an innovative sign function sliding mode reaching law that is driven by a customized LQIR-driven sliding surface. The hybridized control scheme generates optimal control decisions yielded by the LQIR while mimicking the robustness characteristic of SMC against bounded exogenous disturbances. Additionally, the SMC reaching law in the proposed control scheme is augmented with a nonlinear adaptation mechanism that flexibly modulates the control activity to effectively compensate for the external perturbations while minimizing the chattering content. The controller parameters are numerically optimized offline. The efficacy of the prescribed hybrid control law is analyzed via customized MATLAB simulations that normalize the patient’s BG level to 80 mg/dL, under measurement noise and meal disruptions, from an initial hyperglycemic state. The results justify the improved BG regulation accuracy and disturbance-rejection capability of the proposed control procedure.

## 1. Introduction

Type-I Diabetes (TID) is a disorder of the endocrine system, which is characterized by the degeneration of the insulin-generating pancreatic T-cells caused by an autoimmune attack or genetic predisposition [1]. This disorder mitigates the body’s capacity to create the insulin required to maintain the blood glucose (BG) levels in the bloodstream at the desired reference, which subsequently induces metabolic dysregulation [2]. This metabolic disorder has affected the quality of life of millions of people all over the globe [3]. The TID patients rely upon well-postulated BG regulation control of the exogenous insulin administration to normalize their BG levels under every condition to prevent any diabetic complications [4]. Designing a reliable control law for the aforementioned biological endocrine system that maintains reasonable resilience against exogenous glycemic disturbances presents a challenging control problem [5].

### 1.1. Literature review

The Bergman Minimal Model (BMM) is a widely used mathematical representation of glucose-insulin dynamics in T1D patients [6]. This model is essential for understanding how insulin impacts glucose levels in the blood. The Cobelli model is an extension of the Bergman model that incorporates additional physiological processes such as insulin secretion, hepatic glucose production, and peripheral glucose uptake [7]. It is particularly useful for simulating the effects of different insulin therapies. The Hovorka model is a comprehensive mathematical model for glucose-insulin dynamics in TID patients [8]. This model is widely used in the design and testing of closed-loop insulin delivery systems (artificial pancreas). The Sorensen model is a detailed compartmental model that describes glucose and insulin kinetics, incorporating multiple compartments for insulin absorption, distribution, and degradation [9]. The Dalla Man model includes a detailed description of glucose absorption from the gut, insulin secretion, and glucose-insulin interaction [10]. This model accounts for both intravenous and oral glucose tolerance tests, making it suitable for a wide range of simulation scenarios.

In scientific literature, numerous closed-loop BG regulatory control techniques have been put forth to optimize the exogenous insulin administration for glycemic control of TID patients [11]. The pervasive Proportional Integral Derivative (PID) controllers are exceedingly popular for glycemic regulation due to their computational simplicity and reliable control yield [12, 13]. However, they are rendered ineffective against unmodeled nonlinear disturbances owing to their limited degrees of freedom. The augmentation of fractional-order and complex-order calculus tends to increase the resilience and adaptability of the PID control procedure by introducing new hyper-parameters in its structure [14, 15]. However, the offline optimization of these parameters is a laborious task. The neuro-fuzzy intelligent control procedures are known to deliver an agile BG control effort. However, the neural controller requires a large and reliable training data set to synthesize an accurate inverse control law [16]. The fuzzy logic controllers, on the other hand, require an elaborate set of well-postulated qualitative rules that are empirically derived as per the expert’s knowledge [17]. Dependence on an expert’s knowledge inevitably induces imprecision in the empirically derived set of rules. The computational requirements of the aforementioned intelligent control procedure put an excessive burden on the computer.

The Linear-Quadratic-Regulator (LQR) controller is a renowned state feedback compensator that minimizes a pre-configured quadratic performance index of state variables and control inputs to provide optimum control decisions [18]. However, despite its innate optimal behavior, the model-based nature incapacitates the LQR to robustly compensate for the identification errors, model changes, and parametric indeterminacies contributed by the bounded exogenous disturbances [19, 20]. Owing to its versatility and capacity to optimize performance over a prediction horizon, the Model Predictive Control (MPC) has become a robust method for glucose regulation [21]. However, resource-constrained situations may find it difficult to implement MPC since it can be computationally demanding and may require sophisticated algorithms for real-time realization [22].

The ubiquitous Sliding-Mode-Controller (SMC) is a variable-structure compensator that is well-known for its robust control yield [23]. It effectively rejects the parametric variations and external disturbances by efficiently transitioning between the sliding surfaces with the aid of a pre-configured switching function [24]. The SMC scheme, and its modified variants, have been extensively used in the scientific literature to regulate the BG concentration levels in TID patients, especially under meal-induced perturbations [25]. Modified variants of fast terminal sliding mode control strategies are particularly beneficial for under-actuated systems, where precise and rapid control is essential [26, 27]. Despite its inherent robustness, the repetitive switching in the SMC law leads to highly discontinuous control yield that induces chattering in the patient’s BG concentration levels [28]. The resulting ripples in the BG state response are detrimental to the health of the TID patient [29]. The control strategy proposed in [30] attempts to improve chattering in a conventional SMC technique. The integral sliding mode control (ISMC) introduces an integral component in the sliding surface that helps in smoothing the control action, whereas the adaptive backstepping can use the online parameter adaptation to reduce the chattering. Utilization of a high-gain, as well as variable-gain differentiator in the SMC variants, enhances the accuracy of state and derivative estimations under noisy conditions, which ensures superior trajectory tracking and stabilization for underactuated systems [31, 32]. However, the aforementioned approaches put unnecessary computational burdens on the computer.

The H-infinity control can robustly reject exogenous disturbances and parametric uncertainties and disturbances, which makes it an effective tool for glucose regulation applications where patient dynamics and meal disturbances tend to vary randomly [33]. However, it can also be excessively conservative while aiming to minimize the worst-case scenario, which often leads to sub-optimal performances. While backstepping control schemes work very effectively for smooth nonlinearities in BG regulation applications; however, unlike H-infinity, they lack the necessary resilience against large perturbations contributed by meal intake or stress conditions [34]. The ubiquitous adaptive control strategies self-tune the controller parameters online as per the changes in the patient’s state variations [35]. The complexity of adaptive algorithms, however, may potentially result in higher processing demands and possible instability when commuting between various control modes [36]. Recently, machine learning (ML) techniques have gained a lot of traction in enhancing glucose regulation through predictive modeling and clinical decision support [37]. However, despite their effectiveness, the aforementioned techniques necessitate large datasets and intensive training, which inevitably puts excessive recursive computational burden on the computer [38].

The LQR is ideal for applications where an optimal control yield is required while preserving the asymptotic stability of the system; whereas, the SMC laws are best suited for applications where the system is required to robustly nullify the impact of exogenous (bounded) disruptions [39]. The BG-level regulation system has all the aforementioned requirements [40]. It requires the control law to robustly and accurately track the reference BG level, even under the influence of meal disturbances, while guaranteeing an optimal insulin infusion rate (IIR) as well as the system’s closed-loop stability [41].

### 1.2. Proposed methodology

The novel contribution of this paper is to formulate and validate a hybrid robust-optimal BG regulation control procedure for TID patients to normalize their BG concentration levels while effectively rejecting the impact of bounded meal disruptions and sensor noise. The dynamics of the virtual patient are simulated via the well-known Bergman Minimal Model (BMM). The ubiquitous Linear-Quadratic-Integral Regulator (LQIR) is used as the benchmark control procedure for comparison with the proposed scheme. The proposed hybrid control procedure is realized by devising an LQIR-driven self-regulating SMC law. The LQIR’s nominal control input is used to formulate the SMC law that collaborates with the baseline optimal regulator to efficiently reject the bounded meal disruptions while maintaining an economical IIR. The SMC law’s switching gain is dynamically adjusted online via a pre-configured nonlinear hyperbolic adaptation function, which further enhances the reference BG-level tracking and disturbance attenuation ability of the prescribed hybrid control procedure. The innovative contributions of this research work are listed below:

1. Design of a nominal LQIR using the BMM to regulate the BG levels in TID patients.
2. Formulation of an SMC law that uses LQIR’s control input to develop the customized sliding surface.
3. Linear combination of the LQIR with the proposed SMC law to constitute the proposed collaborative LQIR-driven SMC law that beneficially combines the optimality and robustness of its constituent controllers. The stability analysis of the prescribed control procedure is also presented.
4. Self-regulation of the SMC’s switching gain through a customized nonlinear hyperbolic scaling function of the sliding surface.
5. Investigation of the prescribed control procedure via software simulations that validate its setpoint regulation and disturbance rejection capacity.

The proposed innovative LQIR-driven SMC strategy innovatively retrieves the benefits of the aforesaid control schemes while avoiding the inherent problems. The hybridized control approach generates an optimal control yield (synonymous with LQR) while mimicking the robustness characteristic of SMC against bounded meal disruptions. Furthermore, the prescribed control procedure avoids the non-robust reachability phase associated with the ubiquitous SMC law while robustly compensating for matched uncertainties [39]. The stability proof of the prescribed control procedure is also presented. Additionally, the proposed control procedure is also augmented with a sliding surface-dependent nonlinear hyperbolic adaptation function that dynamically increases the SMC switching gain under the influence of meal-induced BG-level disruptions, and vice versa. The said modification also mitigates the chattering in the state responses by generating a relatively smoother control input while preserving its robustness. The formulation and simulation of the proposed LQIR-driven self-regulating SMC law for a BG regulation system has not been investigated in the open literature thus far. Hence, the idea presented in this paper is novel.

### 1.3. Innovative features of the proposed control law

The LQIR-driven SMC with adaptive switching gain offers a powerful combination of optimal control, robustness, and adaptability. It is especially suitable for dynamic environments with high uncertainty, such as the BG regulation problem. Table 1 presents a brief qualitative comparison of the proposed control law with MPC, backstepping, and other controllers. The proposed control scheme ensures fast response and safe insulin administration, with improved disturbance rejection (against meals and stress) due to adaptive gain tuning.

**Table 1:** Comparative analysis of the proposed scheme with other controllers.

Feature	Proposed Scheme	MPC [21]	Adaptive Backstepping [34]	PID [13]	Fuzzy Logic [17]
**Robustness to Uncertainty**	High	Moderate	Moderate	Low	Good
**Optimality**	Optimal	Sub-optimal	Sub-optimal	Low	Sub-optimal
**Response Time**	Fast	Slow	Moderate	Fast	Moderate
**Handling Meal Disturbances**	Effective	Effective	Moderate	Poor	Effective
**Chattering**	Low	None	None	Moderate	Moderate
**Computational Complexity**	Moderate	High	Moderate	Low	High
**Design Complexity**	Moderate	Complex	Challenging	Simple	Moderate
**Handling Nonlinear Dynamics**	Better	Better	Good	Poor	Good
**Safety against Hypoglycemia**	High	Good	Good	Limited	Fair

On the contrary, the MPC offers optimal glucose control but is computationally expensive and highly sensitive to model variations and prediction errors [21]. Due to ream-time gain optimization, it has a slower response time and high computation burden. Adaptive backstepping is effective for nonlinear systems but may struggle with meal disturbances [34]. It shows gradual adaptation to new and unpredictable disturbances (meals or stress activity). It involves recursive tuning and subsystem design, which makes the design complex and increases the computational load. The classical PID controller is easy to implement but lacks robustness [13]. The fuzzy controllers provide good disturbance handling but may not match the precision or the computational economy of LQIR-driven adaptive SMC [17]. Moreover, its robustness and computational burden depend on the number of rules used to implement the control law. Therefore, the LQIR-driven adaptive SMC is a strong candidate for personalized and robust blood glucose management, addressing the key challenges in maintaining safe and stable glycemic levels.

The remainder of the paper is arranged as follows: Section 2 presents the glucose-insulin model and the LQIR’s constitution for BG regulation. Section 3 presents the formation and stability analysis of the proposed LQIR-driven self-regulating SMC law. Section 4 presents the controller parameter tuning procedure. Section 5 examines the outcomes of the software simulations. Section 6 discusses the challenges presented by the practical implementation of the proposed control scheme along with potential future research directions. Section 7 concludes the work.

## 2. Glucose-insulin regulation mechanism

**Fig 1:**
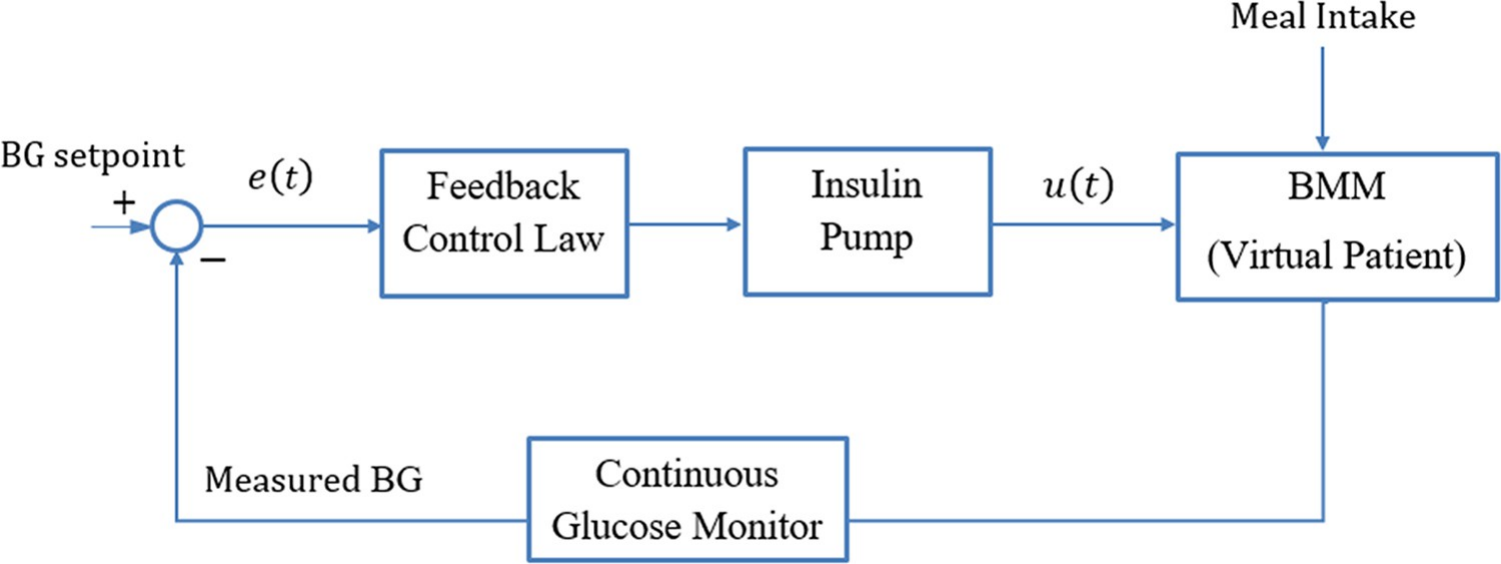
Schematic of the closed-loop glucose-insulin regulation mechanism [15].

This section presents a closed-loop control procedure used for delivering insulin to prevent hyperglycaemia in TID patients [41]. As described earlier, an excessive (or insufficient) insulin infusion alters the patient’s BG concentration levels, which has a negative impact on their health. The BG levels can be normalized by continually infusing appropriate amounts of insulin into the bloodstream of the TID patient. The glucose-insulin regulation schematic is depicted in Fig 1 [15]. As the subcutaneous injection approach mimics the body’s natural insulin release, it is believed to be the safest way to give the body an insulin dose. The schematic comprises a glucose sensor that continuously monitors and compares the BG concentration levels with the setpoint reference (80mg/dL). Based on the instantaneous error in the BG levels, a feedback control procedure is used to actuate an insulin pump that variably modifies the IIR in the patient’s bloodstream. The infusion is stopped when the BG level approaches the setpoint.

### 2.1. Glucose-insulin dynamic model

The patient’s glucose-insulin dynamics are mathematically modeled by using the BMM approach [14]. This technique accurately assesses the functionality of the artificial pancreas and identifies its model while presenting very few biological complications.

While the Hovorka, Cobelli, or Sorensen models for simulating the dynamics of insulin delivery systems offer comprehensive physiological detail, the BMM is chosen in this study owing to its simplified and flexible structure, which allows for easier implementation, adaptation, and interpretation of results [6–10]. The BMM is an excellent tool for demonstrating fundamental glucose-insulin dynamics and developing a reliable control system design without the added complexity of the aforementioned models. For initial proof-of-concept studies, the BMM provides a straightforward framework to test hypotheses and develop BG regulation control algorithms before applying them to more complex models. This approach ensures that basic functionalities are correctly implemented and potential issues are identified early. The computational requirements of BMM aid in performing numerous iterations quickly, facilitating efficient optimization and sensitivity analyses. Additionally, parameter calibration in the BMM is more straightforward due to the smaller number of parameters involved as compared to other sophisticated models, making it suitable for scenarios with limited clinical data.

The glucose metabolism process dictated by the BMM is represented via the following set of differential equations [15, 42].

$$\begin{array}{c} \dot{G}(t)=-p_{1}G(t)-X(t)(G(t)-G_{ss})+\frac{G_{m}(t)}{V_{1}}\\ \dot{X}(t)=-p_{2}X(t)+p_{3}I(t)\\ \begin{array}{c} \dot{I}(t)=-n(I(t)+I_{b})+\frac{v(t)}{V_{1}}\\ \dot{\varepsilon }(t)=G_{ss}-G(t) \end{array} \end{array}\}$$ (1)

where, *G*(*t*) is the BG concentration level, *X*(*t*) is the insulin concentration level in a “remote” compartment, *I*(*t*) is the blood-insulin concentration level, *G*_*m*_(*t*) is the meal disruption input, *v*(*t*) is the modified IIR that serves as the system’s control input, *G*_*ss*_ is the steady-state BG concentration and is treated as the set point (reference) BG-level, *I*_*b*_ is the steady-state concentration level of blood-insulin, *n* is the plasma insulin’s decay-rate, *V*_1_ is the blood volume, *p*_1_ is the rate parameter for glucose effectiveness that quantifies the effectiveness of glucose to be cleared from the bloodstream independent of insulin action, *p*_2_ is the insulin clearance rate that represents how quickly the effect of insulin diminishes after it is released into the bloodstream, and *p*_3_ is the rate parameter for insulin sensitivity that measures the response of glucose uptake by muscle cells to insulin, $\dot{\varepsilon }(t)$ is the error between the patient’s actual and steady-state BG concentration levels, and *ε*(*t*) is the BG error integral variable. The pre-configured model parameters *p*_1_,*p*_2_, and *p*_3_ are associated with the blood specimen being analyzed. The error integral supplements the system’s setpoint-tracking accuracy and strengthens its damping against meal disturbances [43]. The state space representation of the glucose-insulin system model is shown below.

$$\dot{x}(t)=Ax(t)+Bu(t)+Fd(t)+HG_{ss},y(t)=Cx(t)+Du(t)$$ (2)

where, *x*(*t*) is the state vector, *y*(*t*) is the output vector, *u*(*t*) is the control input, *d*(*t*) is the disturbance input, ***A*** is the system matrix, ***B*** is the control input matrix, ***F*** is the disturbance input matrix, ***C*** is the output matrix, and ***D*** is the feed-forward matrix. The state and input vectors of the said system are expressed in Eq 3.


$$x(t)={\left[\begin{array}{ccc} G(t) & X(t) & \begin{array}{cc} I(t) & \varepsilon (t) \end{array} \end{array}\right)}^{T},u(t)=v(t),d(t)=G_{m}(t)$$ (3)

The matrices associated with the system’s state space model are given by Eq 4 [15].


$$A=\left[\begin{array}{ccc} -p_{1} & G_{b} & \begin{array}{cc} 0 & 0 \end{array}\\ 0 & -p_{2} & \begin{array}{cc} p_{3} & 0 \end{array}\\ \begin{array}{c} 0\\ -1 \end{array} & \begin{array}{c} 0\\ 0 \end{array} & \begin{array}{c} \begin{array}{cc} -n & 0 \end{array}\\ \begin{array}{cc} 0 & 0 \end{array} \end{array} \end{array}\right],B=\left[\begin{array}{c} 0\\ 0\\ \begin{array}{c} \frac{1}{V_{1}}\\ 0 \end{array} \end{array}\right],F=\left[\begin{array}{c} \frac{1}{V_{1}}\\ 0\\ \begin{array}{c} 0\\ 0 \end{array} \end{array}\right],H=\left[\begin{array}{c} 1\\ 0\\ \begin{array}{c} 0\\ 1 \end{array} \end{array}\right],$$

$$C=[\begin{array}{ccc} 1 & 0 & \begin{array}{cc} 0 & 0 \end{array} \end{array}],D=[\begin{array}{cc} 0 & 0 \end{array}]$$ (4)

The state variable *G*(*t*) is also designated as the system’s output variable. Table 2 lists the model parameters utilized in this study for a healthy individual and three distinct patients [24].

**Table 2:** Model parameters for a healthy individual and three distinct patients [24].

Parameter	Units	Healthy Individual	Patient 1	Patient 2	Patient 3
*p*_1_	min^−1^	0.0317	0.012	0.011	0.015
*p*_2_	min^−1^	12.3 × 10^−3^	20 × 10^−3^	7.2 × 10^−3^	14.2 × 10^−3^
*p*_3_	min^−1^	4.92 × 10^−6^	5.3 × 10^−6^	2.16 × 10^−6^	99.4 × 10^−6^
*n*	min^−1^	0.2659	0.3	0.2465	0.2814
*I*_*b*_	mU/L	7	7	7	7
*G*_*ss*_	mg/dL	80	80	80	80
*V*_1_	L	12	12	12	12
*G*(0)	mg/dL	200	200	200	200
*X*(0)	min^−1^	0	0	0	0
*I*(0)	mU/L	50	50	50	50

### 2.2. Baseline LQIR formulation

The LQIR is an optimal state space control strategy that includes an auxiliary integral error state variable to improve the setpoint-tracking accuracy and damping against overshoots and undershoots [44].

An energy-like quadratic performance index (QPI) of the input and state variables is minimized. The Hamilton-Jacobi-Bellman (HJB) equations are then solved to devise the aforementioned optimal state-compensator offline [45]. The QPI used here is expressed below.

$$S_{lq}=\frac{1}{2}\int_{0}^{\infty }\left({x(t)}^{T}Qx(t)+{u(t)}^{T}Ru(t)\right)dt$$ (5)

where ***R*** ∈ ℝ is a preset positive definite control penalty matrix and ***Q*** ∈ ℝ^4×4^ is a preset positive semi-definite state penalty matrix. The ***Q*** and ***R*** matrices, respectively, govern input variations and penalize the state of the system. The following is a symbol representation of these matrices.


$$Q=diag(\begin{array}{cc} \begin{array}{cc} q_{G} & q_{X} \end{array} & \begin{array}{cc} q_{I} & q_{\varepsilon } \end{array} \end{array}),R=\rho$$ (6)

The coefficients of these matrices are denoted as *q*_*x*_≥0 and *ρ*>0. These coefficients are adjusted offline through the tuning process covered in Section 4. The following algebraic Riccati equation (ARE) uses the optimized set of aforementioned coefficients to calculate its solution, the ***P*** matrix.

$$A^{T}P+PA-PBR^{-1}B^{T}P+Q=0$$ (7)

where ***P*** is a positive definite symmetric matrix with dimensions of ℝ^4×4^. The state compensator gain vector ***K*** is computed as indicated in (8).

$$K=R^{-1}B^{T}P$$ (8)

where, ***K*** = [*k*_*G*_ *k*_*X*_ *k*_*I*_ *k*_*ε*_]. The nominal LQIR law is expressed as follows.


$$u_{lq}(t)=-Kx(t)$$ (9)

**Fig 2:**
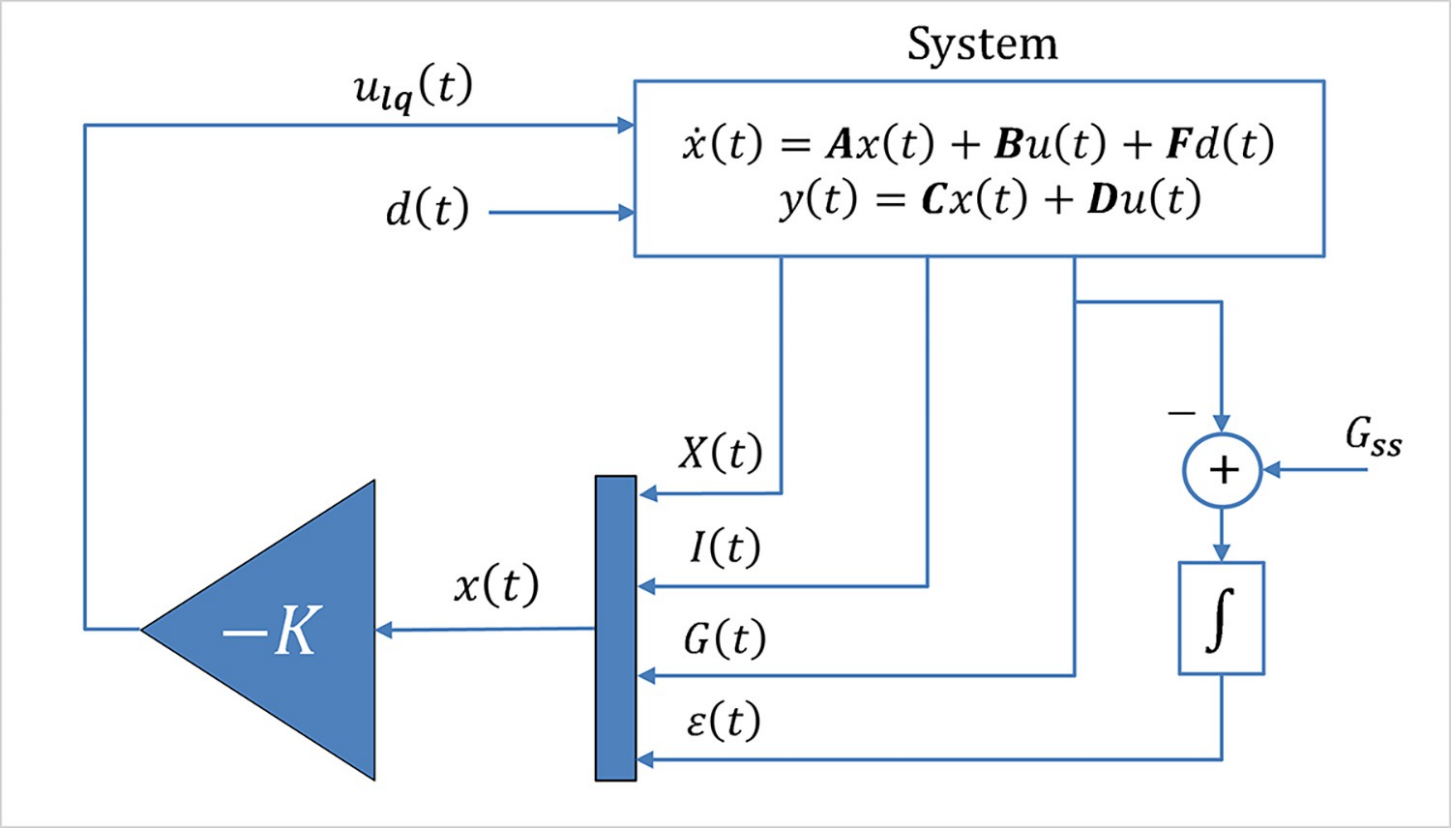
Block diagram of the nominal LQIR law.

Fig 2 displays the block diagram of the LQIR scheme.

The LQIR’s convergence is investigated via the following Lyapunov function [46].


$$W(t)={x(t)}^{T}Px(t)>0,forx(t)\neq 0$$ (10)

The first derivative of *W*(*t*) is derived, as shown below.


$$\dot{W}(t)=2{x(t)}^{T}P\dot{x}(t)$$

$$=2{x(t)}^{T}P(A-BK)x(t)$$

$$=2{x(t)}^{T}P(A-BR^{-1}B^{T}P)x(t)$$

$$={x(t)}^{T}(PA+A^{T}P)x(t)-2{x(t)}^{T}(PBR^{-1}B^{T}P)x(t)$$ (11)

By substituting Eq (7) in (11), $\dot{W}(t)$ is simplified as shown in (12).


$$\dot{W}(t)=-{x(t)}^{T}Qx(t)-{x(t)}^{T}(PBR^{-1}B^{T}P)x(t)<0$$ (12)

If ***R*** = ***R***^*T*^>0 and ***Q*** = ***Q***^*T*^≥0, then $\dot{W}(t)$ is a negative-definite function. The designed LQIR law’s asymptotic stability can thus be preserved by meeting the aforementioned conditions.

## 3. Proposed control methodology

To counteract the effects of parametric uncertainties and input disturbance, the designed LQIR is integrated with a discontinuous SMC to constitute the proposed hybrid control procedure. The sliding surface of the SMC is thus formulated as a function of the LQIR control input *u*_*lq*_(*t*). Once designed, the LQIR-driven SMC is augmented with an online adaptive function that dynamically adjusts its switching gain as an even-symmetric hyperbolic scaling function of the BG-level error, $\dot{\varepsilon }(t)$. The formulation of the prescribed hybrid BG controller and its stability study are presented in the following section.

### 3.1. Hybrid LQIR-based SMC law

The system’s linear state space equation provides information regarding meal disturbance and reference BG concentration. To derive the proposed SMC law, the following state equation is considered.

$$\dot{x}(t)=Ax(t)+Bu(t)+Fd(t)+HG_{ss}$$ (13)

where *d*(*t*) is the disturbance input with some known maximum value. The hybrid (collaborative) control law is expressed as follows [39].

$$u(t)=u_{lq}(t)+mu_{s}(t)$$ (14)

where *u*_*S*_(*t*) represents the sliding-mode control input and *m* is a real-numbered scaling factor. Inserting the aforementioned control input in the system’s state equation yields the following expression.


$$\dot{x}(t)=Ax(t)+Bu_{lq}(t)+Bmu_{s}(t)+Fd(t)+HG_{ss}$$ (15)

The SMC law thus utilizes the following sliding surface [25].


s(t)=Gx(t)+z(t) (16)

where ***G*** is an empirically-defined positive state-weighting vector of the form ***G*** = [*g*_1_ *g*_2_ *g*_3_ *g*_4_], and *z*(*t*) is new variable that depends on the system dynamics. The coefficients of the ***G*** vector are adjusted offline through the tuning process covered in Section 4. The derivative of *s*(*t*) is presented as follows.


$$\dot{s}(t)=G\dot{x}(t)+\dot{z}(t)$$ (17)

It is desired for $s(t)=\dot{s}(t)=0$ during sliding operation. The substitution of Eq (15) in (17) delivers the following expression.


$$\dot{s}(t)=G(Ax(t)+Bu_{lq}(t)+Bmu_{s}(t)+Fd(t)+HG_{ss})+\dot{z}(t)=0$$ (18)

The equation can be simplified as follows.


$$\dot{s}(t)=G(Ax(t)+Bu_{lq}(t)+HG_{ss})+GBmu_{s}(t)+GFd(t)+\dot{z}(t)=0$$ (19)

It is to be noted that GB=g3V1 and GF=g1V1. Hence, if the value of m=g1g3, then $\dot{s}(t)$ can be written as shown in (20).


$$\dot{s}(t)=G(Ax(t)+Bu_{lq}(t)+HG_{ss})+\frac{g_{1}}{V_{1}}u_{s}(t)+\frac{g_{1}}{V_{1}}d(t)+\dot{z}(t)=0$$ (20)

During the sliding phase, the SMC signal *u*_*S*_(*t*) = −*d*(*t*) eliminates the bounded exogenous disturbance(s). Thus, the variable $\dot{z}(t)$ is expressed as shown below.

$$\dot{z}(t)=-G(Ax(t)+Bu_{lq}(t)+HG_{ss}),z(0)=-Gx(0)$$ (21)

where *x*(0) = [200 0 50 0]^*T*^ as listed in Table 1. As a result of the substitution, m=g1g3, it can be concluded that the effect of matched uncertainty has been eliminated and, thus, the system is directed by the nominal control input *u*_*lq*_(*t*) provided by the LQIR. The insertion of $\dot{z}(t)$ in (19) guarantees the following simplification of $\dot{s}(t)$ [39].


$$\dot{s}(t)=GBmu_{s}(t)+GFd(t)$$ (22)

The integration of $\dot{z}(t)$ delivers the following expression.


$$-z(t)=z(0)-G\int_{0}^{t}\left(Ax(\tau )+Bu_{lq}(\tau )+HG_{ss}\right)d\tau$$ (23)

Hence, the final expression of the sliding surface *s*(*t*) is expressed in (24), [39].


$$s(t)=G\left(x(t)-x(0)-\int_{0}^{t}\left(Ax(\tau )+Bu_{lq}(\tau )+HG_{ss}\right)d\tau \right)$$ (24)

#### a. Lyapunov stability proof

Once the sliding surface *s*(*t*) is evaluated, the SMC law *u*_*S*_(*t*) is derived using the Lyapunov theorem. The following positive Lyapunov function is utilized for this purpose.


$$Y(t)=\frac{1}{2}s^{T}(t)s(t)$$ (25)

The Lyapunov function’s first derivative is expressed in (26).


$$\dot{Y}(t)=s^{T}(t)\dot{s}(t)$$ (26)

The substitution of Eq (32) in (36) yields the following expression.


$$\dot{Y}(t)=s^{T}(t)GBmu_{s}(t)+s^{T}(t)GFd(t)$$ (27)

The SMC law in (28) is used to fulfill the Lyapunov stability criteria, $\dot{Y}(t)<0$, [39].

$$u_{s}(t)=-\beta sgn\left(s(t)\right)$$ (28)

where *β* is the switching gain of the control law, and sgn(.) is the signum function expressed in (29).


$$sgn(s(t))=\left\{ \begin{array}{l} -1,\;ifs(t)<0\\ 0,\;ifs(t)=0\\ 1,\;ifs(t)<0 \end{array}\right.$$ (29)

Inserting the SMC law in (27) delivers the modified expression of $\dot{Y}(t)$.


$$\dot{Y}(t)=-GBm\beta \;s(t)+s^{T}(t)GFd(t)$$

=s(t)(−GBmβ+GFd(t)) (30)

As discussed earlier, the $\dot{Y}(t)<0$ for system to be asymptotically stable. This stability criterion implies that,

−GBmβ+GFd(t)<0 (31)

The substitutions B=g3V1,GF=g1V1, and m=g1g3 in (31), deliver the following condition.


β>d(t) (32)

The condition in (32) preserves the closed-loop stability of the SMC scheme. If the vector ***G*** and the parameters *m* and *β* are selected such that the conditions GB=g3V1,GF=g1V1,m=g1g3, and *β*>*d*(*t*) are satisfied, the time derivative of the Lyapunov function becomes negative definite, that is, $\dot{Y}(t)<0$. This guarantees that the system will converge to the sliding surface asymptotically, ensuring robust stability.

In lieu of the stability condition in (40), the value of *β* is adjusted offline through the tuning process discussed in Section 4. The aforementioned condition states that the modulation gain *β* must be chosen larger than the maximum bound of the disturbance *d*(*t*) to maintain stability. In this study, the control input disturbances are bounded in the range 0 to 4 mU/min. This range is empirically selected based on the pilot simulations. The objective is to avoid abrupt large IIR requirements, which may cause discomfort to patients due to the switching behavior of the controller’s SMC component. This range can be altered by the users as per the requirement of the application. Consequently, the value of *β* is chosen from the range 4 and 10 mU/min. The upper limit of *β* is set at10 mU/min to avoid an aggressive control approach, which may eventually lead to hypoglycemia or constantly fluctuating BG levels.

#### b. Control law formulation

**Fig 3:**
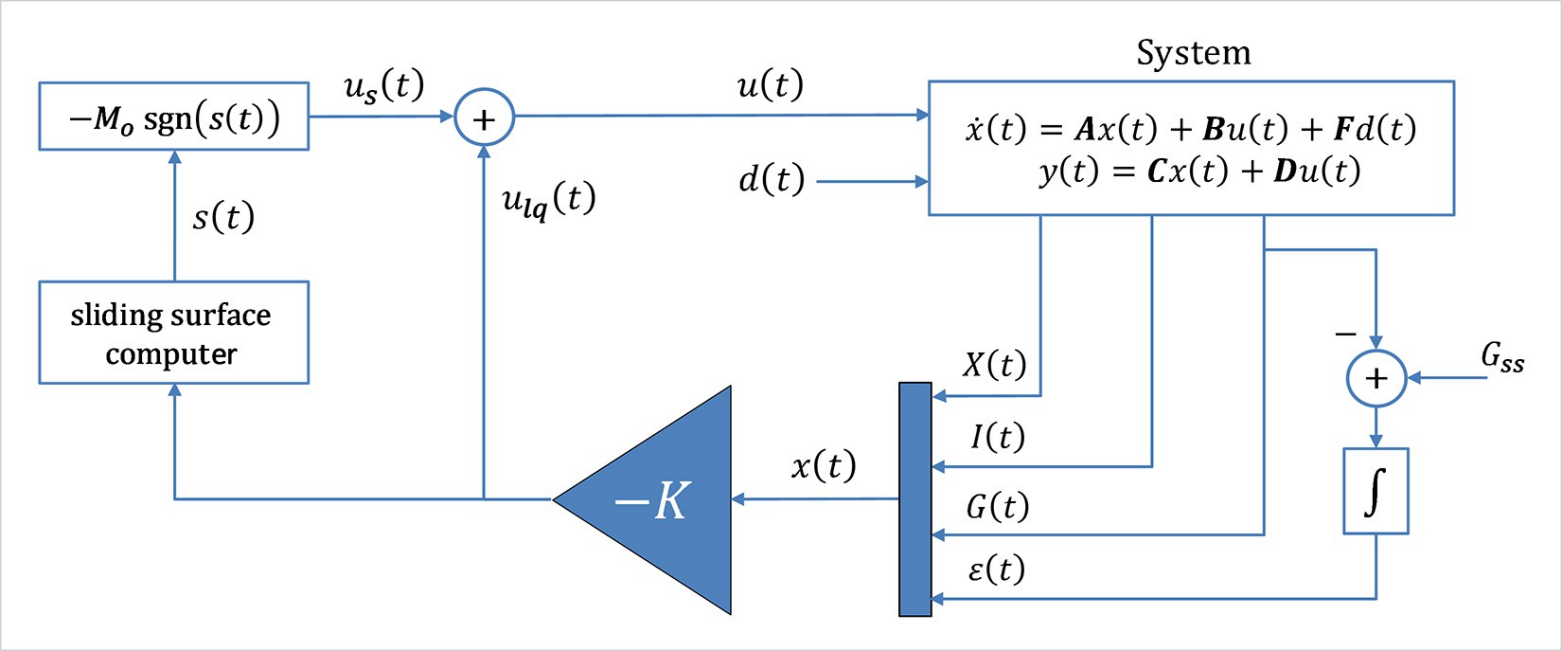
Block diagram of the nominal LQ-SMC law.

The hybrid LQIR-driven SMC law, as suggested in (14), is formulated as follows.

$$u(t)=-Kx(t)-M_{o}sgn(s(t))$$ (33)

where, *M*_*o*_ = *mβ*. The said control law is referred to as the LQ-SMC law in the following sections. Fig 3 displays the block diagram of the LQ-SMC scheme.

### 3.2. Hybrid LQIR-based self-adaptive SMC law

To minimize the chatter in the state response, imposed by the hard limiter, the sgn(*s*(*t*)) function is replaced by a relatively smoother nonlinear function tanh(*s*(*t*)) [47]. The modified hybrid control law is expressed, as shown in (34).


$$u(t)=-Kx(t)-M_{o}tanh(s(t))$$ (34)

It is to be noted that the SMC law is majorly needed under external disturbances and parametric disturbances [48]. However, chattering is inexorably introduced into the state response due to the disruptive control input contributed by the SMC scheme. Therefore, the control law in (33) is also augmented with an error-dependent adaptation tool that increases the SMC’s adaptability by dynamically adjusting its switching gain as the BG levels vary. The following rationale is thus adopted:

1. Under large BG concentration level errors (hyperglycemia state), the SMC’s switching gain is inflated to strongly damp the overshoots in the BG level and quickly restore it to the steady-state value.
2. Under small BG concentration level errors (equilibrium state), the SMC’s switching gain is reduced to maintain the BG level at the steady-state value with minimal fluctuations.

These properties speed up the transition of the BG level from the state of hyperglycemia to the normal level and strengthen the closed-loop system’s damping against meal disturbances while economizing the application of the control input (the IIR) [49]. The aforesaid adaptation strategy is realized by augmenting the said control law with a nonlinear gain scaling function. The rationale requires the scaling function to be bounded, differentiable, smooth, and even symmetric.

The hyperbolic secant function (HSF) yield a smooth and gradual gain adjustment which aids in stabilizing the state behavior with minimal chattering [50]. However, it requires calculation of exponentials in both directions. This can be computationally demanding, though modern processors handle these efficiently. The hyperbolic tangent functions exhibit smooth yet faster transitions, leading to a good chattering suppression with a risk of overshoot [47]. However, it can be realized by evaluating a single exponential, making it relatively faster than HSF. The sigmoid functions yield smooth gain adaptation but can introduce slower convergence compared to HSF, causing residual chattering [51]. Its computational complexity is at par with HSF. The exponential functions, although simplest to compute, but its fast decay risks under-damping the system if not tuned properly, causing loss of control effectiveness [52]. Hence, based on the qualitative analysis, the HSF driven by the BG-level error $\dot{\varepsilon }(t)$ is chosen for gain adjustment in this study. The HSF offers a balanced gain adjustment that avoids saturation issues and smoothly regulates the gain under disturbances, enhancing stability for complex systems, without sacrificing control authority. The merits of each of the aforementioned nonlinear functions are summarized in Table 3.

**Table 3:** Comparative analysis of HSF with other nonlinear functions.

Nonlinear Function	Stability	Chattering Mitigation	Computational Complexity
Hyperbolic Secant [50]	High	Excellent	Moderate
Hyperbolic Tangent [47]	Moderate	Good	Low
Sigmoid [51]	Moderate	Fair	Moderate
Exponential [52]	Fast	Good	Low

The comparative analysis in the Table 3 suggests that HSF offers a compelling trade-off between stability, chattering reduction, and computational cost. Hence, in this study, the HSF is adopted to adjust the switching gain *M*(*t*) as a nonlinear function of the BG-level error $\dot{\varepsilon }(t)$. The formulation is shown in (35), [50].

$$M(t)=M_{o}(1-sech(\gamma \;\dot{\varepsilon }(t)))$$ (35)

where, sech(.) Represent the HSF, and *γ* represent the variation rate of the HSF. The proposed hybrid LQIR-driven self-adaptive SMC law is expressed in (36).


u(t)=−Kx(t)−M(t)tanh(s(t)) (36)

**Fig 4:**
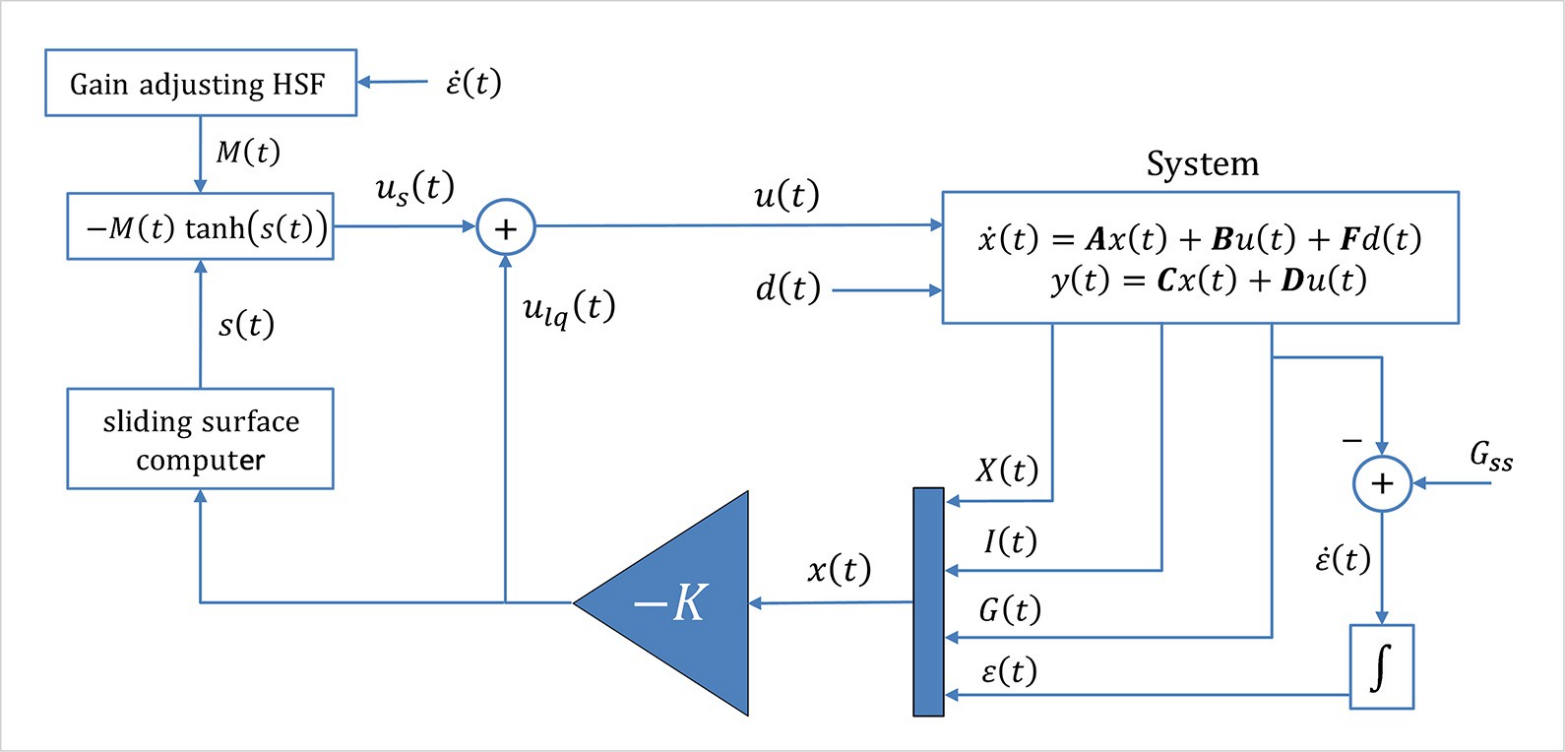
Block diagram of the nominal LQ-ASMC law.

The proposed hybrid control law mentioned above is referred to as LQ-ASMC in the article. Fig 4 displays the block diagram of the LQ-ASMC scheme.

The proposed controller, and its variants, detect the disturbance by monitoring deviations of the system state *G*(*t*) (patient’s BG level) from the desired setpoint BG level, denoted as $\dot{\varepsilon }(t)$. These measurements are fed to both the LQIR and SMC controllers. A significant deviation suggests that the nominal LQIR controller might be insufficient, requiring the SMC to take over.

The system uses nominal LQIR inputs in tandem with the SMC’s corrective actions. The LQIR is used for optimal control during nominal conditions, ensuring minimal energy consumption and smooth system behavior. Under disturbance conditions, as the error magnitude continues to inflate, the SMC action is activated and progressively strengthened. Hence, instead of abrupt switching, the proposed LQ-ASMC ensures continuous blending of control effort via a smooth nonlinear scaling function driven by the magnitude of state error ($\dot{\varepsilon }(t))$. The modulation gain *M*(*t*) of the control law in (36) is dynamically adjusted, ensuring that the SMC contribution only activates and amplifies when needed to counteract disturbances without causing overcompensation or oscillations (chattering). Hence, the LQIR continues operating to maintain performance in every condition, whereas, the SMC compensates for uncertainties by providing corrective control inputs based on the sliding surface. The SMC component cancels out disturbances by compensating with an input equal to the disturbance but opposite in direction.

By initializing the sliding surface from the start (*s*(*t*) = 0), the SMC avoids the unstable reachability phase, ensuring stability from *t* = 0. The discontinuous signum function with a smoother approximation function of the form tanh(*s*(*t*)), as shown in (34) and (36). This softens the switching action, reducing high-frequency oscillations (chattering) while maintaining robustness.

### 3.3. Implementation scheme of the proposed control law

The methodological steps used for the execution and implementation of the LQ-ASMC law are given as follows:

#### 1. Identify Model

- Define the state variables, *x*(*t*).
- Define the differential equations dictating the glucose-insulin dynamics.
- Define the state space matrices (**A**,**B**,**C**,**D**) of the glucose-insulin model.

#### 2. Design LQR

- Define state and control weighting matrices (***Q*** and ***R***).
- Solve the Riccati equation to find the optimal feedback gain matrix ***K***.
- Compute the control input using the LQR.

#### 3. Design ASMC

- Compute the sliding surface *s*(*t*) using the current states, desired states, and LQR control input.
- Define the sliding mode reaching law, M(t) tanh(*s*(*t*)).
- Formulate a nonlinear scaling function to online adapt the scaling gain, *M*(*t*).
- Calculate the SMC input.

**4. Formulate the LQ-ASMC law by combining LQR and ASMC law.**
**5. Implement the control algorithm**

- Continuously acquire the system’s current states.
- Compute the updated LQR control input.
- Compute the sliding surface, *s*(*t*)
- Modify the sliding-mode gain, *M*(*t*).
- Calculate the final control input using the LQ-ASMC law.
- Apply the control input to the system.
- Update the system state variables based on dynamics.

**Fig 5:**
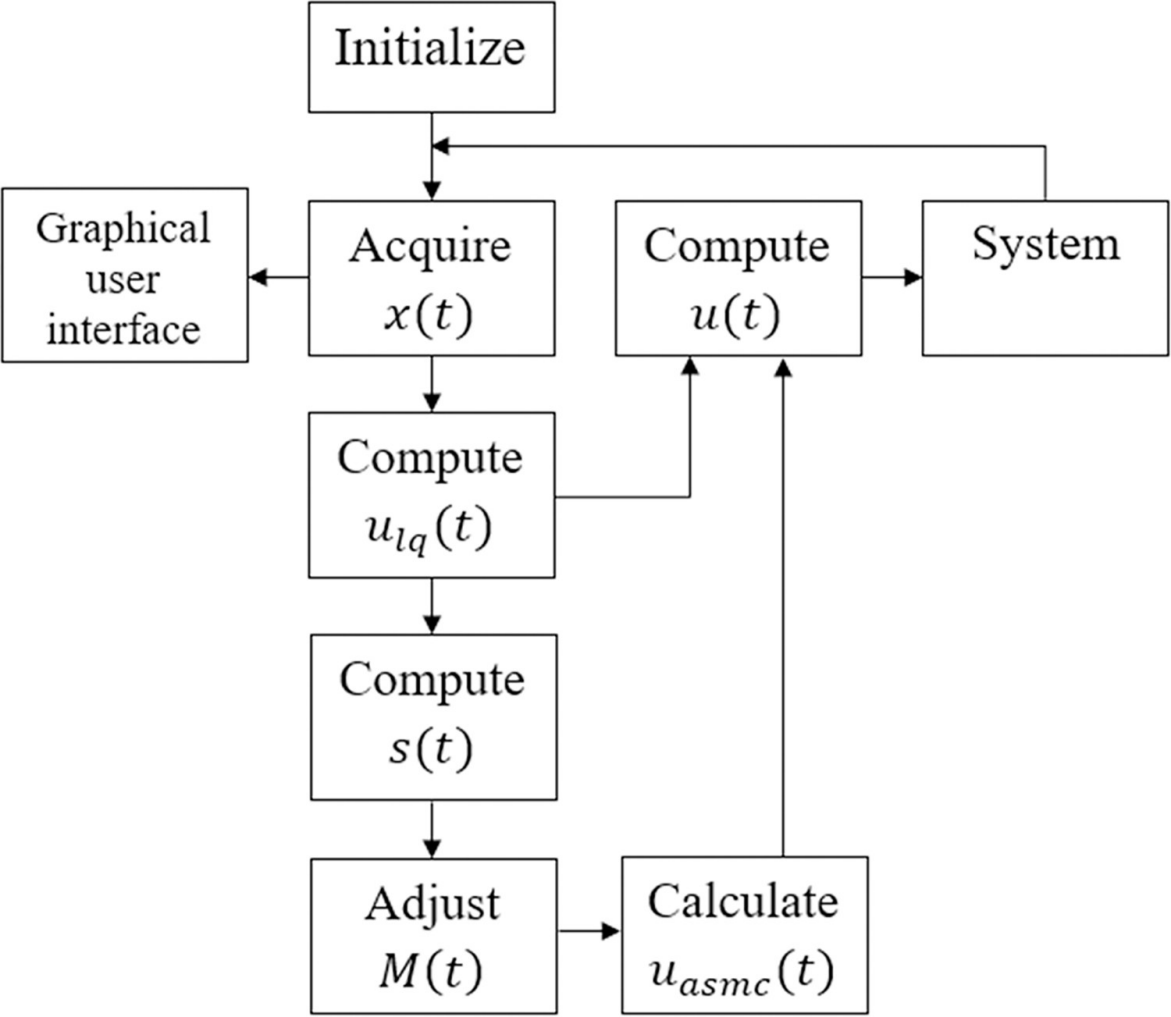
Flow chart of the proposed control scheme.

The flow chart depicting the algorithm implementation of the LQ-ASMC is shown in Fig 5.

## 4. Parameter tuning procedure

The LQIR design is dependent on the state of the system and changes in control input. To guarantee an optimal control effort, it is crucial to allocate the proper weights to the control input and state variables [53]. In lieu of these conditions, the empirical settings of the ***Q*** matrix, ***R*** matrix, ***G*** vector, and other parameters (*β*,*m*, and *γ*) are limited by the designer’s knowledge and thus, accurate reference tracking and optimal transient recovery behavior might not always be achieved [54, 55].

As derived in (12), the state weighting and control weighting matrices are selected such ***R*** = ***R***^*T*^>0 and ***Q*** = ***Q***^*T*^≥0 to ensure the Lyapunov stability of the LQIR law. Similarly, as derived in (32), the value of *β* is selected as *β*>*d*(*t*) to ensure the Lyapunov stability of the proposed SMC law.

### 4.1. Offline tuning procedure

**Fig 6:**
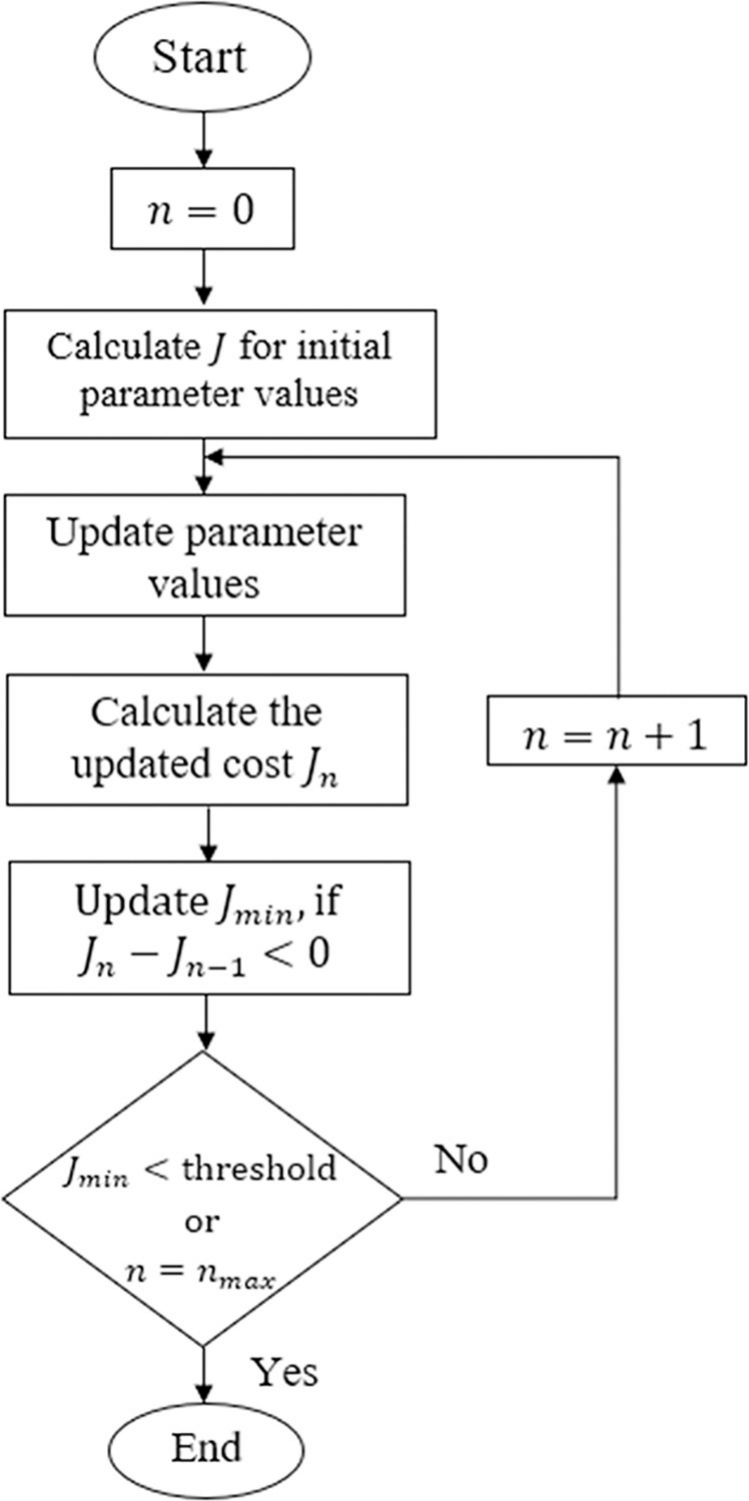
Flow chart of the parameter tuning algorithm [10].

The aforementioned parameters are optimized offline by minimizing a new objective function that captures the variations in BG concentration level error $\dot{\varepsilon }(t)$ and control input *u*(*t*). This objective function is expressed in (37).

$$J=|T_{set}|+\int_{0}^{\infty }\left({\left|\dot{\varepsilon }(t)\right|}^{2}+{\left|u(t)\right|}^{2}\right)dt$$ (37)

where, *T*_*set*_ is the time taken by the BG level to stabilize within ±5% of *G*_*ref*_. To deliver optimum control decisions, the function *J* imposes equal weight on the two minimization criteria. The initial values of these parameters can be chosen at random from the aforementioned search space. The algorithm then coordinates the exploration in the direction of the descending slope of *J* [56]. The parameter optimization scheme is demonstrated in Fig 6 [15]. The BMM parameters of Patient 1 (See Table 1) serve as the benchmark for the tuning purpose. The simulations discussed in Section 4 are used for parameter tuning. In every tuning attempt, the controller parameters are adjusted based on pre-defined increments. Upon the re-adjustment of the parameters, the control law is commissioned to normalize the virtual patient’s BG concentration levels at 80 mg/dL from an initial value of 200 mg/dL for 300 min using the refined set of parameters. Once the trial is concluded, the cost *J*_*n*_ is calculated; where, *n* is the trial number. In the event that the current trial’s cost (*J*_*n*_) turns out to be less than the previous trial’s cost (*J*_*n*−1_), the local minimum cost *J*_*min*_ is modified. This configuration guarantees that the search is moving in the descending gradient direction of *J*. The exploration for the optimum parameter values is concluded if either *J*_*min*_ reaches a predetermined setpoint cost or the algorithm has completed the maximum number of trials (*n*_*max*_) permitted [56]. The setpoint for *J*_*min*_ is set at 1×10^7^ in this work [10]. Because each simulation takes a long time, the value of *n*_*max*_ is fixed at 8. The selected parameters are further refined by manually adjusting their values.

### 4.2. Parametrization of control law

For designing the baseline LQIR, the coefficients of ***Q*** and ***R*** matrices are tuned from the range [0, 1]. The tuning process begins with ***R*** = 1 and ***Q*** = diag(1 1 1 1). The coefficients thus tuned via the afore-described tuning procedure are $q_{G}=0.32,q_{I}=0.22,q_{X}=0.25,q_{\varepsilon }=0.61$, and *ρ* = 1.05. The gain vector ***K*** = [*k*_*G*_ *k*_*X*_ *k*_*I*_ *k*_*ε*_], linked with the LQIR law *u*_*lq*_(*t*) = −***K****x*(*t*), is computed using the tuned set of ***Q*** and ***R*** matrices. The state-compensator gains thus evaluated are $k_{G}=0.0038,k_{X}=0.0021,k_{I}=0.0024$, and *k*_*ε*_ = 1.12×10^−4^.

For designing the LQ-SMC law, the coefficients of the ***G*** vector are picked from the range [0, 0.01]. The tuning process begins with ***G*** = [0.01 0.01 0.01 0.01]. The coefficients of the vector ***G*** = [*g*_1_ *g*_2_ *g*_3_ *g*_4_] thus chosen are $g_{1}=0.0088,g_{2}=0.0051,g_{3}=0.0052,g_{4}=3.65\times 10^{-4}$. Consequently, the value of *m* is computed as 1.59. As discussed in Section 3.1 (a), the value of *β*>*d*(*t*) is selected from the range [4, 10]. The initial value of *β* is 4.0. The value of *β* yielded by the tuning procedure for this study is 4.48. Hence, the value of *M*_*o*_ = *mβ* is computed as 7.12. The LQ-SMC law is given as follows.


u(t)=−Kx(t)−7.12tanh(s(t)) (38)

For designing the LQ-ASMC law, the variation rate of the HSF is picked from the range [0, 1]. The tuning process begins with an initial value of *γ* = 0.1. The value of *γ* thus chosen for this research is 0.06. The LQ-ASMC law is given as follows.


$$u(t)=-Kx(t)-7.12\left(1-sech(0.06\dot{\varepsilon }(t))\right)tanh\left(s(t)\right)$$ (39)

## 5. Simulations and analysis

Clinical trials are avoided at this stage because conducting experiments involving human subjects requires extensive ethical approval processes, significant financial resources, and logistical resources. Instead, this article outlines a phased approach, where successful simulation results will serve as the basis for experimental validation in future works. The focus of this paper on establishing the effectiveness and reliability of the controller through rigorous simulation studies is both appropriate and necessary given the preliminary nature of this study. The simulations allow to iteratively refine and optimize the controller design, which validates the framework before committing to the more resource-intensive process of human trials, thereby ensuring a higher likelihood of success in subsequent phases. This approach aligns with standard research methodologies, where simulation results guide the design and implementation of human trials.

The test simulations undertaken to verify the time optimality and robustness of the prescribed BG control strategies are presented in this section, along with a comparative analysis of the attained results.

### 5.1. Simulation setup

The time-domain behavior of the LQIR, LQ-SMC, and LQ-ASMC schemes are compared through customized simulations that test the controller’s behavior under hyperglycaemia state, transient meal disturbance, and Gaussian noise. The MATLAB/Simulink R2020b software tool is used to implement the control application and run the simulations [57]. A 64-bit embedded computer with a 2.1 GHz CPU and 12.0 GB RAM is used to run the software. The samples are acquired after every 60 seconds. As mentioned in Section 2, three distinct BMMs are used to simulate the virtual TID patients (See Table 1) to validate the efficacies of designed control laws in effectively regulating the BG concentration levels from an initial state of hyperglycaemia as well as rejecting the transient meal disturbance and measurement (Gaussian) noise. To avoid hypoglycemia in the patients due to a significant drop in BG levels, the IIR signal *u*(*t*) is limited between 0 mU/min and 100 mU/min.

### 5.2. Simulation results

The following two testing scenarios are employed to benchmark the proposed LQ-ASMC against the nominal LQIR and the LQ-SMC. The simulations are used to test the behavior of the three controllers individually on each patient. The closed-loop system is responsible for continuously tracking the patient’s BG level to 80 mg/dL setpoint under every operating condition. In every test case, white Gaussian noise is added to *G*(*t*) to observe the impact of random sensor noise on the reference tracking behavior.

#### A. BG regulation under hyperglycaemia

**Fig 7:**
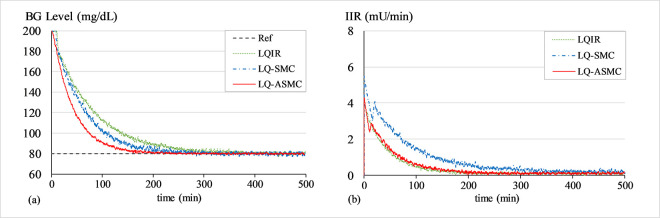
(a) BG levels of Patient 1 under hyperglycaemia, (b) IIR (control input) for Patient 1 under hyperglycaemia.

**Fig 8:**
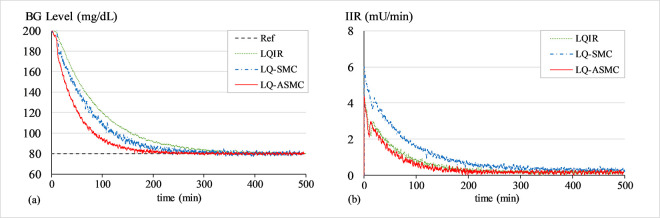
(a) BG levels of Patient 2 under hyperglycaemia, (b) IIR (control input) for Patient 2 under hyperglycaemia.

**Fig 9:**
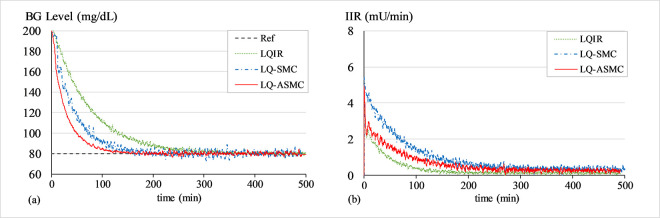
(a) BG levels of Patient 3 under hyperglycaemia, (b) IIR (control input) for Patient 3 under hyperglycaemia.

The purpose of this simulation is to test the control law’s ability to bring the patients’ BG concentration levels down from an initial state of hyperglycaemia (200 mg/dL) to 80 mg/dL. At the start of every trial, a white Gaussian noise signal with a mean of zero and variance of 0.2 is added to *G*(*t*) to simulate the effects of measurement noise from the glucose sensor. Figs 7–9 show the BG level and IIR (control input) profiles under LQIR, LQ-SMC, and LQ-ASMC for Patients 1, 2, and 3, respectively.

#### B. BG regulation under transient meal disruption

**Fig 10:**
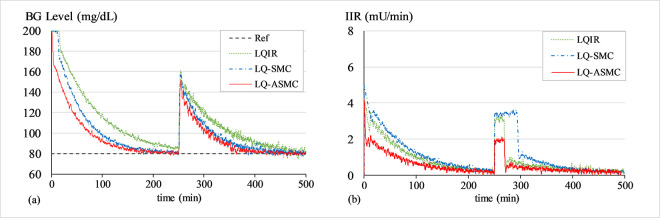
(a) BG levels of Patient 1 under meal disturbance, (b) IIR (control input) for Patient 1 under meal disturbance.

**Fig 11:**
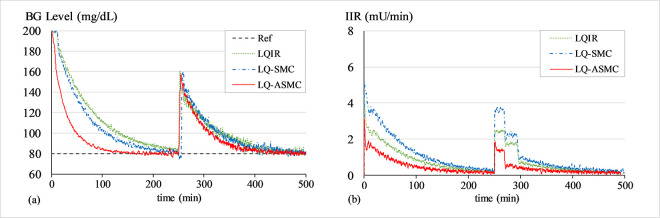
(a) BG levels of Patient 2 under meal disturbance, (b) IIR (control input) for Patient 2 under meal disturbance.

**Fig 12:**
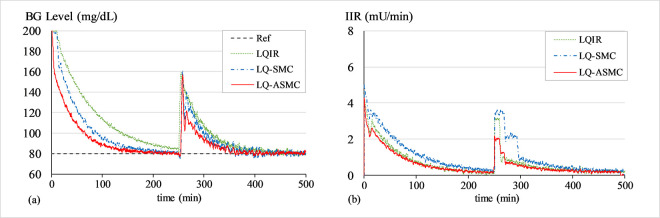
(a) BG levels of Patient 3 under meal disturbance, (b) IIR (control input) for Patient 3 under meal disturbance.

The purpose of this simulation is to evaluate the control law’s robustness against bounded exogenous disturbances, which are typically brought on by the consumption of food. A simulated impulse signal in *d*(*t*) and a white Gaussian noise signal in *G*(*t*) are used to simulate the effects of meal disruption and sensor noise, respectively. The closed-loop system operates to recover from the transient perturbation and normalizes the patient’s BG levels back to 80 mg/dL baseline after the said disruption. A white noise signal with a mean of zero and variance of 0.2 is introduced into the system at the start of the trial, while a simulated impulse signal with an amplitude of 80 mg/dL is injected into the system at t ≈ 500 min. Figs 10–12 show the BG level and IIR (control input) profiles under LQIR, LQ-SMC, and LQ-ASMC for Patients 1, 2, and 3, respectively.

#### C. BG regulation under physical or mental stress

**Fig 13:**
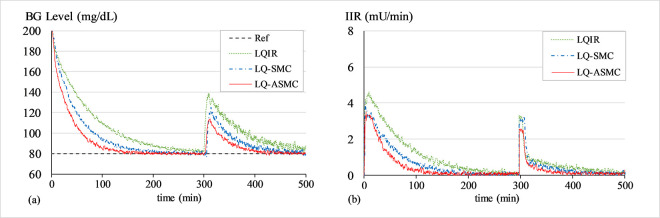
(a) BG levels of Patient 1 under stress, (b) IIR (control input) for Patient 1 under stress.

**Fig 14:**
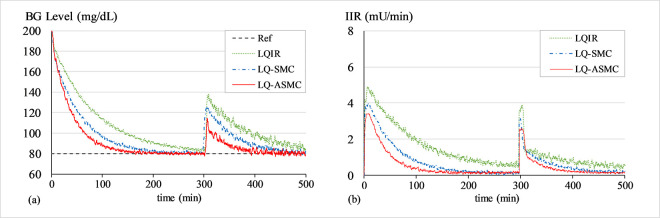
(a) BG levels of Patient 2 under stress, (b) IIR (control input) for Patient 2 under stress.

**Fig 15:**
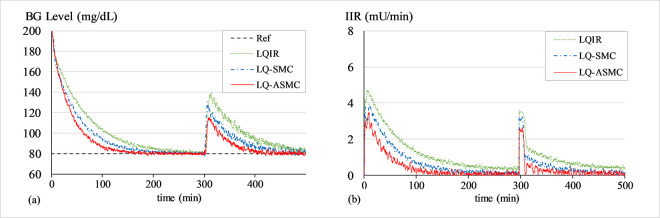
(a) BG levels of Patient 3 under stress, (b) IIR (control input) for Patient 3 under stress.

The purpose of this simulation is to assess the controller’s robustness against unprecedented variations in physiological model parameters, which are typically brought on by physical or mental stress. To emulate stress conditions, the parameter *p*_1_ is decreased to reflect reduced glucose uptake by muscles, parameter *p*_2_ is increased to simulate increased hepatic glucose output triggered by stress hormones, and parameter *p*_3_ is decreased to account for reduced insulin effectiveness due to the release of the counter-regulatory hormones like cortisol and adrenaline into the blood. These hormones typically cause the BG levels to rise. To carry out this simulation, the values of *p*_1_,*p*_2_, and *p*_3_ are changed to $8.0\times 10^{-3}{min}^{-1},25.0\times 10^{-3}{min}^{-1}$, and 2.5×10^−6^min^−1^ at t ≈ 500 min, respectively. This modification changes the coefficients of the system matrix ***A*** presented in (4), which causes the BG levels to rise. Figs 13–15 show the BG level and IIR (control input) profiles under LQIR, LQ-SMC, and LQ-ASMC for Patients 1, 2, and 3, respectively.

### 5.3. Analysis and discussions

The following Critical Performance Measures (CPMs) are employed to analyze the simulation results.

- E_rms_: Root-mean-squared magnitude of the BG-level error $\dot{\varepsilon }(t)$.
- E_sa_: Sum of the absolute magnitudes of the BG-level errors $\dot{\varepsilon }(t)$.
- T_fall_: Time needed by the BG level to fall to +10% of *G*_*ref*_ from the hyperglycaemia.
- T_set_: Time needed by the BG level to stabilize within ±5% of *G*_*ref*_.
- T_rec_: Time needed by the BG level to stabilize after a transient disturbance.
- OS: The magnitude of overshoot observed in BG level after a transient disturbance.
- U_ms_: Mean-squared magnitude of the IIR input, *u*(*t*).
- U_p,start_: Peak magnitude of IIR input during the initial hyperglycemic state.
- U_p,dist_: Peak magnitude of IIR input during meal disturbance.

The E_rms_ is calculated as shown below.

$$E_{rms}=\sqrt{\frac{1}{N}\sum_{n=0}^{N}{\left|\dot{\varepsilon }(n)\right|}^{2}}$$ (40)

where, *N* is the total number of samples and *n* is the number of measurements. The value of *N* is 500 for simulation A and 1000 for simulation B in this article. The E_sa_ is calculated, as shown below.


$$E_{sa}=\sum_{n=0}^{N}\left|\dot{\varepsilon }(n)\right|$$ (41)

The U_MS_ is calculated, as shown below.


$$U_{ms}=\frac{1}{N}\sum_{n=0}^{N}{\left|u(n)\right|}^{2}$$ (42)

A quantitative analysis of the robustness and performance of the prescribed BG control strategies is conducted using the afore-described CPMs. Table 4 summarizes the simulation outcomes of all test cases for Patient 1. Table 5 presents the simulation outcomes of all test cases for Patient 2. Table 6 summarizes the simulation outcomes for Patient 3. The outcomes confirm the enhanced response speed and disturbance compensation ability of the LQ-ASMC law over the LQIR and LQ-SMC laws.

**Table 4:** Simulation results of Patient 1.

Simulation	CPM		Control Law		
	Metric	Unit	LQIR	LQ-SMC	LQ-ASMC
A	E_rms_	mg/dL	33.98	31.13	25.64
	E_sa_	mg/dL	1367.43	1255.52	759.88
	T_fall_	min.	162	130	110
	T_set_	min.	225	177	145
	U_ms_	(mU/min)^2^	4.72	6.08	3.44
	U_p,start_	mU/min	4.33	5.47	4.22
B	E_rms_	mg/dL	41.79	34.20	26.18
	E_sa_	mg/dL	2451.77	1680.33	1247.56
	T_rec_	min.	190	168	161.5
	U_ms_	(mU/min)^2^	6.31	9.59	4.54
	U_p,dist_	mU/min	3.33	3.62	2.32
C	E_rms_	mg/dL	34.72	27.51	23.46
	E_sa_	mg/dL	2053.75	1291.08	945.98
	OS	mg/dL	58.73	45.32	35.02
	T_rec_	min.	185	145	112
	U_ms_	(mU/min)^2^	5.16	4.88	3.83
	U_p,dist_	mU/min	4.62	4.05	3.02

**Table 5:** Simulation results of Patient 2.

Simulation	CPM		Control Law		
	Metric	Unit	LQIR	LQ-SMC	LQ-ASMC
A	E_rms_	mg/dL	38.22	34.11	27.25
	E_sa_	mg/dL	1832.57	1456.16	829.95
	T_fall_	min.	199	161	115
	T_set_	min.	241	208	157
	U_ms_	(mU/min)^2^	4.53	7.35	3.82
	U_p,start_	mU/min	4.39	6.01	4.38
B	E_rms_	mg/dL	38.25	35.84	25.41
	E_sa_	mg/dL	2191.52	1801.85	1231.17
	T_rec_	min.	178	174	163
	U_ms_	(mU/min)^2^	4.98	8.76	2.52
	U_p,dist_	mU/min	2.51	3.76	1.85
C	E_rms_	mg/dL	36.08	27.66	22.21
	E_sa_	mg/dL	2232.03	1407.29	878.91
	OS	mg/dL	58.31	44.45	33.86
	T_rec_	min.	196	152	103
	U_ms_	(mU/min)^2^	6.73	5.21	4.74
	U_p,dist_	mU/min	4.91	3.96	3.41

**Table 6:** Simulation results of Patient 3.

Simulation	CPM		Control Law		
	Metric	Unit	LQIR	LQ-SMC	LQ-ASMC
A	E_rms_	mg/dL	34.02	26.09	24.88
	E_sa_	mg/dL	1526.31	1277.92	813.45
	T_fall_	min.	182	115	85
	T_set_	min.	226	148	101
	U_ms_	(mU/min)^2^	3.31	6.13	4.05
	U_p,start_	mU/min	4.40	5.52	4.95
B	E_rms_	mg/dL	36.38	28.67	21.05
	E_sa_	mg/dL	2130.87	1609.17	1468.93
	T_rec_	min.	138	134	131
	U_ms_	(mU/min)^2^	4.75	8.07	3.47
	U_p,dist_	mU/min	3.18	3.61	2.24
C	E_rms_	mg/dL	31.72	26.37	23.22
	E_sa_	mg/dL	1785.28	1278.5	916.91
	OS	mg/dL	59.55	50.54	34.24
	T_rec_	min.	178	149	107
	U_ms_	(mU/min)^2^	4.98	4.59	3.99
	U_p,dist_	mU/min	4.71	3.95	3.48

In **Simulation A**, every controller displays a distinct transient reaction to bring every patient’s BG levels to the setpoint BG concentration level. The LQIR exhibits a comparatively longer settling time to converge to *G*_*ss*_ and a mediocre reference tracking accuracy. The LQ-SMC shows a significant improvement in reference-tracking behavior over the LQIR due to its faster response time and more accurate regulation. The LQ-ASMC law exhibits an improved setpoint-tracking accuracy and the fastest transient response. It efficiently suppresses the steady-state fluctuations and promptly stabilizes the BG concentration levels at the intended setpoint, all the while subsidizing the IIR expenditure.

In **Simulation B**, each control law applies appropriate resources to normalize the BG concentration levels after the simulated meal disturbance has induced a state of hyperglycaemia in the virtual patient. The LQIR lacks the resilience to withstand sensor noise and meal disturbances. The time domain profile under LQIR manifests a slower transient recovery time and comparatively more chattering is displayed in the response. Moreover, the LQIR injects insulin for a longer time period, which is not practical from a therapeutic standpoint for the patients.

The LQ-SMC exhibits a significantly improved disturbance rejection capacity and a relatively faster error convergence rate. However, it also results in a substantial requirement for insulin infusion. With a notable increase in resilience against meal disturbances and measurement noise, the LQ-ASMC law exhibits the most time-optimal behavior. It displays minimum-time transient recovery, minimal setpoint-tracking error, and comparatively faster response speed. Finally, it reduces the unnecessary ripples in state responses by generating a relatively cheaper and smoother control input (IIR) profile.

In **Simulation C**, the patient experiences hyperglycemia due to the stress conditions simulated by appropriately varying the model parameters. The LQIR exhibits fragile control behavior against the stress-induced perturbations, exhibiting a large overshoot and slowest transient recovery period. The LQ-SMC manifests a comparatively faster error convergence rate together with a markedly enhanced capacity to reject disturbances. However, it achieves this behavior at the expense of a sustainable amount of insulin infusion. The LQ-ASMC law demonstrates the fastest transient reaction and robust setpoint tracking. It quickly stabilizes the BG levels at the desired setpoint and effectively attenuates the stress-induced overshoot in the response, all while offsetting the IIR consumption.

The LQ-ASMC yields a mean improvement of 20.5%, 40.4%, 30.1%, 15.2%, 30.6%, 26.1%, and 2.5%, in the E_rms_, E_sa_, T_fall_, T_rec_, T_set_, U_ms_, and U_p_, in comparison to the LQIR. The enhanced flexibility of the proposed LQ-ASMC, resulting from the augmentation of the LQIR with the self-adaptive SMC law, is credited with significantly improving the transient reaction speed and the control input efficiency. Finally, the LQ-ASMC yields a mean improvement of 13.6% and 18.8% in the root-mean-square value of the steady state fluctuations (chattering content), in comparison to the LQIR and LQ-SMC, respectively.

The proposed modifications systematically enhance the controller’s self-reasoning to quickly adapt and, thus, execute better setpoint-tracking and disturbance compensation capacity. Moreover, the LQ-ASMC configuration accomplishes the prescribed BG regulation objectives without sacrificing the control input (IIR) economy, which is indeed a noteworthy achievement. While the offline adjustment of a multitude of parameters associated with the LQ-ASMC is a cumbersome process, the procedure’s improved time optimality outweighs this drawback.

### 5.4. Statistical analysis of simulation results

In this section, the data acquired via the simulations is statistically analyzed to justify the aforementioned claims of performance improvement. The BG regulation data of each patient for each distinct control scheme under simulations A, B, and C is examined via the following two data analysis techniques:

- Confidence Interval (CI)
- Hypothesis Testing

#### a. Confidence Interval (CI)

The CIs are used to analyze the BG regulation data to attain an intuitive understanding of the variability and reliability of the results. It helps quantify the degree of uncertainty in the estimated BG levels. A 95% CI, as used in this study, indicates that there is a 95% chance that the true average BG level falls within the determined range. The statistical analysis of the BG regulation data of each patient for each distinct control scheme under simulations A, B, and C, is presented in Table 7. The statistical analysis validates the enhanced performance improvement contributed by LQ-ASMC in normalizing the virtual patient’s BG levels under every testing scenario.

**Table 7:** Statistical data analysis.

Test	Statistical Tool (mg/dL)	Patient 1			Patient 2			Patient 3		
		LQIR	LQ-SMC	LQ-ASMC	LQIR	LQ-SMC	LQ-ASMC	LQIR	LQ-SMC	LQ-ASMC
A	Mean	98.71	94.70	90.36	101.97	97.31	91.61	98.19	90.53	86.68
	Median	84.69	81.82	80.64	86.81	82.78	80.96	84.29	81.83	80.64
	Standard Dev.	28.38	27.44	23.46	31.28	29.40	24.65	28.73	23.89	18.73
	CI (95%)	1.11	1.08	0.92	1.22	1.16	0.97	1.13	0.94	0.73
	Upper CI (95%)	99.82	95.78	91.28	103.19	98.47	92.58	99.32	91.47	87.41
	Lower CI (95%)	97.60	93.63	89.44	100.75	96.15	90.64	97.06	89.58	85.94
B	Mean	110.29	100.77	95.37	106.27	102.95	93.49	105.49	97.11	91.49
	Median	100.21	88.77	85.07	96.02	91.34	83.51	92.74	85.79	83.05
	Standard Dev.	28.80	27.18	21.19	27.81	27.53	21.54	29.33	25.47	18.82
	CI (95%)	1.13	1.06	0.83	1.09	1.08	0.84	1.15	0.99	0.74
	Upper CI (95%)	111.42	101.83	96.20	107.36	104.03	94.34	106.64	98.10	92.23
	Lower CI (95%)	109.16	99.70	94.54	105.18	101.87	92.65	104.34	96.11	90.76
C	Mean	104.63	95.38	91.06	106.77	96.85	90.35	101.43	95.25	90.75
	Median	94.77	85.70	82.31	98.03	87.19	82.26	91.76	85.81	82.41
	Standard Dev.	24.46	22.80	20.70	24.19	21.94	19.65	23.38	21.52	20.59
	CI (95%)	1.36	1.27	1.15	1.34	1.22	1.09	1.30	1.19	1.14
	Upper CI (95%)	105.99	98.65	92.21	108.11	98.06	91.44	102.73	96.44	91.89
	Lower CI (95%)	103.27	94.12	89.91	105.42	95.63	89.26	100.14	94.06	89.61

#### b. Hypothesis testing

The hypothesis testing is done to analyze the effectiveness of the proposed LQ-ASMC procedure in regulating the BG levels, as compared to LQIR and LQ-SMC schemes. It involves testing an assumption (the hypothesis) about the BG levels of patients under the influence of the proposed control law. In this study, two cases of hypothesis testing are carried out to compare the performance of LQ-ASMC against LQIR as well as the performance of LQ-ASMC against LQ-SMC.

*Case 1*: *Null hypothesis (H*_*0*_*)*. The LQ-ASMC procedure does not significantly affect BG regulation compared to the LQIR.

*Alternative hypothesis (H*_*1*_*)*. The LQ-ASMC procedure significantly improves BG regulation compared to the LQIR. The hypothesis examination for Case 1 is done via t-test using the sample data sets of LQIR and LQ-ASMC. The value of significance-level is set to 0.05 for this test. The t-test results are quantified in Table 8. In every patient’s case, the value of the t-stat is larger than the corresponding t-critical value. Hence, hypothesis H_0_ is rejected, and it is concluded that LQ-ASMC is comprehensively better than LQIR. Additionally, it is to be noted that the p-value is lesser than the significance level (0.05) in every case, which supports the decision to reject H_0_ and support H_1_.

**Table 8:** Results of t-test conducted on sample data of LQ-ASMC against LQIR.

Simulation	Statistical Tool (mg/dL)	Patient 1	Patient 2	Patient 3
A	t-stat	11.32	12.99	16.79
	t-critical	1.96	1.96	1.96
	p-value	2.3×10^−29^	6.0×10^−38^	2.5×10^−61^
B	t-stat	20.87	18.17	20.08
	t-critical	1.96	1.96	1.96
	p-value	1.7×10^−92^	2.3×10^−71^	8.9×10^−86^
C	t-stat	14.98	18.64	12.12
	t-critical	1.96	1.96	1.96
	p-value	1.5×10^−48^	1.5×10^−72^	6.8×10^−33^

*Case 2*: *Null hypothesis (H*_*0*_*)*. The LQ-ASMC procedure does not significantly affect BG regulation compared to the LQ-SMC procedure.

*Alternative hypothesis (H*_*1*_*)*. The LQ-ASMC procedure significantly improves BG regulation compared to the LQ-SMC procedure.

The hypothesis testing is done via t-test using the data sets of LQ-SMC and LQ-ASMC. The value of significance-level is set to 0.05. The consequent t-test results are quantified in Table 9. In every patient’s case, the value of the t-stat is larger than the corresponding t-critical value. Hence, hypothesis H_0_ is rejected, and it is concluded that LQ-ASMC is significantly better than LQ-SMC under stress. The p-value < significance level in every case, supporting the decision to reject H_0_ in favor of H_1_.

**Table 9:** Results of t-test conducted on sample data of LQ-ASMC against LQIR.

Simulation	Statistical Tool (mg/dL)	Patient 1	Patient 2	Patient 3
A	t-stat	6.01	7.39	6.31
	t-critical	1.96	1.96	1.96
	p-value	2.0×10^−9^	1.7×10^−13^	3.0×10^−10^
B	t-stat	7.83	13.53	8.86
	t-critical	1.96	1.96	1.96
	p-value	5.9×10^−15^	5.6×10^−41^	1.1×10^−18^
C	t-stat	4.96	7.80	5.34
	t-critical	1.96	1.96	1.96
	p-value	7.4×10^−7^	9.4×10^−15^	1.0×10^−7^

### 5.5. Comparison with modern controllers

To verify the efficacy of the recommended LQ-ASMC controller in handling the BG regulation problems, its performance is also benchmarked against the modern controllers proposed in [15, 24]. These modern control techniques include the complex-order PID (CO-PID) controller [15], fractional-order SMC (FOSMC) [24], adaptive fractional-order SMC (AFOSMC) [24], higher-order SMC (HOSMC) [24], and super-twisting sliding-mode-controller (STSMC) [24]. The simulations A and B in [15, 24] were conducted using the afore-said controllers on the identical trio of patient models, as indicated in Table 2. The performance comparison of the simulation outcomes of each controller is quantified in terms of E_sa_ in Table 10.

**Table 10:** Comparison with modern control techniques.

Simulation	Metric	Unit	Controllers	Patient 1	Patient 2	Patient 3
A	E_sa_	mg/dL	STSMC [17]	1956	2110	1880
			HOSMC [17]	1456	1357	1609
			FOSMC [17]	1135	916	1542
			AFOSMC [17]	877	759	1015
			CO-PID [10]	789	811	855
			LQ-ASMC **(proposed)**	760	829	813
B	E_sa_	mg/dL	STSMC [17]	2720	2986	3560
			HOSMC [17]	2014	1895	2398
			FOSMC [17]	1766	1660	2228
			AFOSMC [17]	1417	1206	1655
			CO-PID [10]	1253	1277	1592
			LQ-ASMC **(proposed)**	1248	1231	1469

The findings in [15] show that the CO-PID controller is designated as the best choice for BG regulation applications. In Simulation A, the LQ-ASMC improves the E_sa_ measure of Patients 1, 2, and 3 by 3.6%, -2.2%, and 4.9%, respectively, in comparison to the CO-PID controller. In Simulation B, the LQ-ASMC improves the E_sa_ measure of Patients 1, 2, and 3 by 0.4%, 3.6%, and 8.3%, respectively, in comparison to the CO-PID controller.

### 5.6. Sensitivity and robustness analysis of LQ-ASMC

This section discusses additional test cases to analyze the sensitivity of LQ-ASMC under the parameter variations as well as its robustness under sensor noise models.

#### a. Sensitivity analysis of LQ-ASMC under variations of all parameters

**Fig 16:**
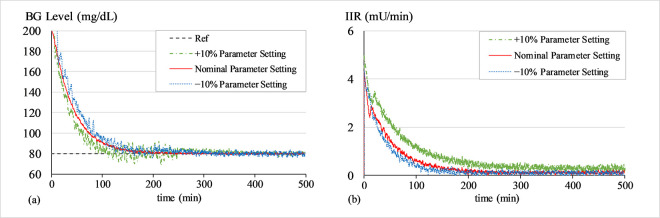
(a) BG levels of Patient 1 under different parameter settings, (b) IIR (control input) for Patient 1 under different parameter settings.

**Fig 17:**
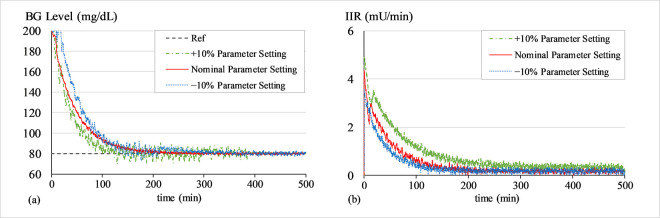
(a) BG levels of Patient 2 under different parameter settings, (b) IIR (control input) for Patient 2 under different parameter settings.

**Fig 18:**
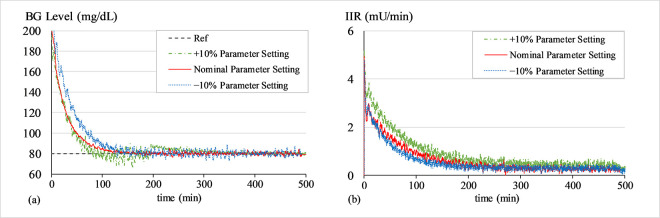
(a) BG levels of Patient 3 under different parameter settings, (b) IIR (control input) for Patient 3 under different parameter settings.

To quantify the robustness of the system regulated by LQ-ASMC to parameter variations and to ascertain the effectiveness of the tuning process in Section 4.1, a detailed sensitivity analysis is performed by introducing a 10.0% increment as well as a 10.0% decrement in the tuned parameter set (***Q***,***R***,***G***,*m*,*β* and *γ*) that is presented in Section 4.2. For each modified parameter set, simulations are conducted to normalize the virtual patient’s BG concentration levels at 80 mg/dL from an initial value of 200 mg/dL for 500 min. The resulting performance metrics are recorded and the consequent results are statistically analyzed to quantify the impact of each parameter on the system’s time-domain performance. Figs 16–18 show the BG level and IIR (control input) profiles with LQ-ASMC under different parameter sets for Patients 1, 2, and 3, respectively. The analysis of the simulation outcomes is quantified in Table 11.

**Table 11:** Sensitivity analysis of LQ-ASMC under different parameter settings.

Metric	Unit	Parameter changes (%)	Patient 1	Patient 2	Patient 3
E_rms_	mg/dL	+10.0	23.37	24.42	20.88
		0	25.64	27.25	24.88
		−10.0	29.54	32.92	26.67
E_sa_	mg/dL	+10.0	815.55	854.28	721.03
		0	759.88	829.95	813.45
		−10.0	1098.96	1237.35	1231.15
T_fall_	min.	+10.0	88	92	78
		0	110	115	85
		−10.0	122	118	105
T_set_	min.	+10.0	133	141	95
		0	145	157	101
		−10.0	152	165	114
U_ms_	(mU/min)^2^	+10.0	3.40	3.55	3.98
		0	3.44	3.82	4.05
		−10.0	12.10	12.18	11.84
U_p,start_	mU/min	+10.0	3.96	3.50	4.76
		0	4.22	4.38	4.95
		−10.0	5.02	4.98	5.21

The parameter settings with a 10.0% increment yield a faster response speed but also inject chattering in the response. The amount of insulin consumed is also relatively higher than the other parameter settings. The parameter settings with a 10.0% decrement yield a relatively slower response speed. The amount of insulin consumed is relatively lesser than the other parameter settings.

The results suggest that the nominal set of parameters (as prescribed in Section 4.2) manifests the best performance and insulin consumption rate. It yields a time-optimal response and accurate reference tracking ability. The nominal set of parameters also avoids causing hypoglycemia in the patients while preserving the IIR efficiency.

#### b. Sensitivity analysis of LQ-ASMC under variations of *β*

**Fig 19:**
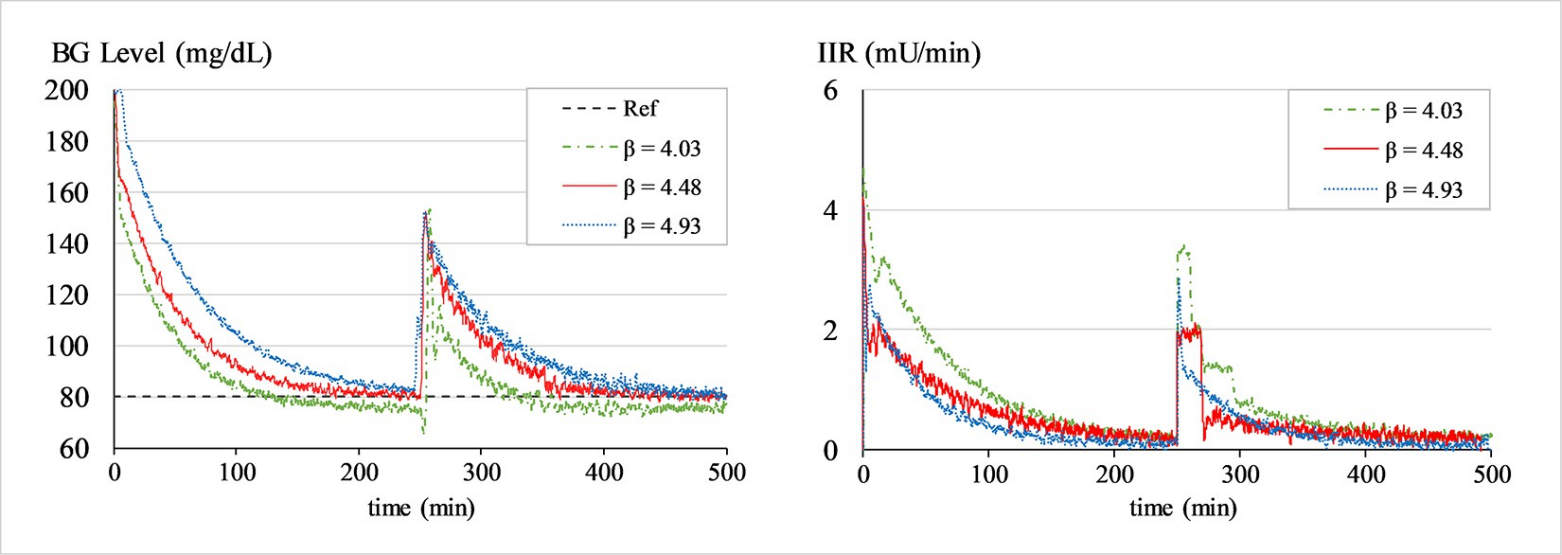
BG levels of Patient 1 under different settings of *β*.

An additional sensitivity analysis is carried out by introducing a 10.0% increment and a 10.0% decrement in the tuned value of *β* only. This simulation is done to measure the resilience of the LQ-ASMC to changes in the *β*. For each modified parameter setting, simulations are conducted to normalize the BG levels of Patient 1 (See Table 2) at 80 mg/dL from an initial value of 200 mg/dL for 500 min. The BG level profiles are recorded and the results are examined to quantify the impact of changes in *β* on the system’s time-domain performance. Fig 19 shows the BG profiles with LQ-ASMC under different settings of *β* for Patient 1. The simulation results are summarized in Table 12. The parameter setting with a 10.0% increment (*β* = 4.93) delivers a relatively aggressive control effort, causing a faster BG convergence rate with a constant offset below the BG setpoint. The parameter settings with a 10.0% decrement (*β* = 4.03) yield an over-damped response. The results suggest that the nominal value of *β* yields a reasonable convergence while damping the steady state fluctuations.

**Table 12:** Sensitivity analysis of LQ-ASMC under different settings of *β*.

**Change in β (%)**	**CPMs of Patient 1 under LQ-ASMC**					
	E_rms_ (mg/dL)	E_sa_ (mg/dL)	T_set_ (min.)	T_rec_ (min.)	U_ms_ (mU/min)^2^	U_p,dist_ (mU/min)
+10.0	24.87	1234.74	132	147	8.73	3.41
0	26.18	1247.56	145	161.5	4.54	2.32
−10.0	32.65	1589.33	156	167.5	2.92	2.87

#### c. Robustness analysis of LQ-ASMC under sensor drift and bias

This customized simulation is performed to test the impact of sensor drift and bias in the proposed BG regulation control system. The sensor’s constant bias refers to a persistent offset in sensor measurements, causing a systematic error in glucose readings. It typically models the calibration errors or misalignment of a sensor. The sensor’s drift is a gradual deviation in sensor readings over time. This phenomenon typically misleads the controller about the actual BG levels. The sensor bias is simulated by adding a fixed offset of 5 mg/dL in the BG measurements. The long-term degradation in the sensor’s accuracy (drift) is simulated by adding a low-frequency sinusoidal signal, having an amplitude of 2 mg/dL and a frequency of 0.2 rad/s, in the BG measurements. The noise signal, presented in (43), is injected into the BG measurements *G*(*t*) to conduct this simulation.


$$G_{n}(t)=5+2\sin\left(0.2t\right)$$ (43)

**Fig 20:**
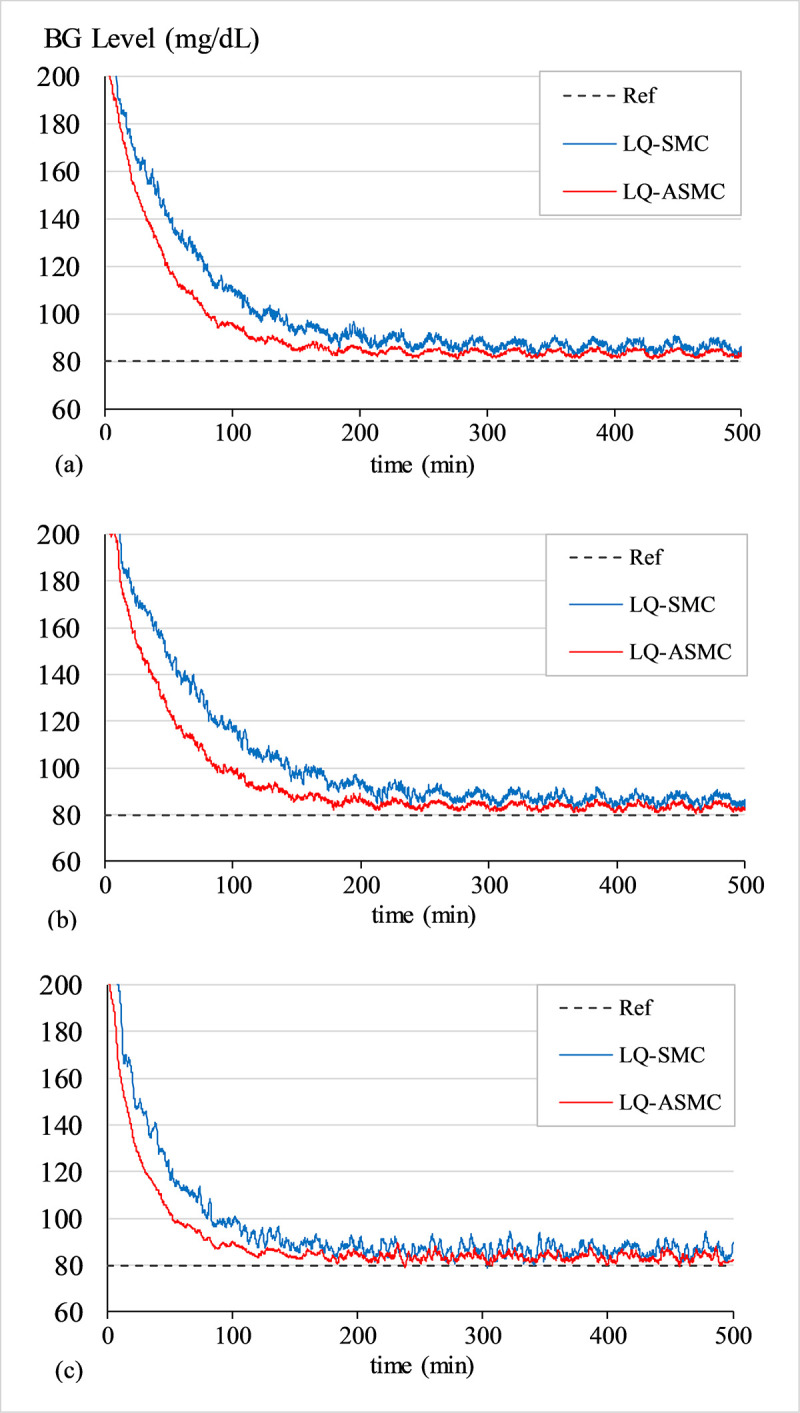
BG levels of (a) Patient 1 under sensor drift and bias, (b) Patient 2 under sensor drift and bias, (c) Patient 3 under sensor drift and bias.

The BG profiles of patients 1, 2, and 3 for LQ-ASMC and LQ-SMC controllers, under the influence of the noise signal *G*_*n*_(*t*), are shown in Fig 20. The consequent simulation results are quantified in Table 13.

**Table 13:** Robustness analysis controllers under sensor drift and bias.

**Patient**	**CPM**		**Control Law**	
	**Metric**	**Unit**	**LQ-SMC**	**LQ-ASMC**
Patient 1	E_rms_	mg/dL	34.67	27.26
	E_sa_	mg/dL	1513.13	1155.26
	T_fall_	min.	148	110
	T_set_	min.	185	147
Patient 2	E_rms_	mg/dL	37.71	28.83
	E_sa_	mg/dL	1691.94	1256.50
	T_fall_	min.	155	116
	T_set_	min.	201	159
Patient 3	E_rms_	mg/dL	28.52	21.28
	E_sa_	mg/dL	1179.56	849.06
	T_fall_	min.	117	88
	T_set_	min.	146	111

### 5.7. Computational requirements

The proposed control algorithm requires dedicated computational resources for the offline calculation of the LQIR gain vector as well as the online computation of the proposed SMC signal at each time step. The latter includes computational resources needed for the online modification of the SMC gain via the hyperbolic function as well. The computation time for each of these control laws is calculated using the built-in ‘tic’ and ‘toc’ commands of MATLAB.

In this study, the LQR gain ***K*** is calculated offline and stored in memory. This reduces the real-time computations to matrix-vector multiplication, *u*_*lq*_(*t*) = −***K****x*(*t*), which has a computational complexity of *O*(*n*^2^), where *n* is the number of state variables. The LQIR gain computation took ~122 μs and used 1.5 MB. However, this computation is done offline and does not contribute to real-time processing. While implementing the self-adaptive SMC component of the proposed control law, the overall complexity of matrix-vector multiplications and numerical integration involved in computing the sliding surface *s*(*t*) is also *O*(*n*^2^). The computation of the adaptive gain function renders a complexity *O*(1) and a computation time of 55.6 μs. The complexity of tanh(.) limiter function is *O*(1) and its computation took 48.6 μs. The average computation time per step taken to calculate the signal generated by the proposed LQIR-ASMC scheme was 45.2 μs with minimal memory overhead. The complexity of this scalar operation is *O*(1).

The memory usage analysis indicates that the proposed control algorithm’s memory footprint is within acceptable limits for real-time applications. The aforementioned profiling results also validate the efficient computation times and low memory overhead for both the LQR computation and the proposed SMC control law. This makes the algorithm suitable for practical implementation in resource-constrained environments.

The real-time implementation of the proposed control scheme can be potentially optimized via the following techniques:

1. To simplify the system while maintaining its fundamental dynamics, model order reduction through balanced truncation can be implemented.
2. The system’s order can be reduced by using fewer state variables and targeting only the essential ones. This will in turn reduce the overall complexity of the system.
3. An even symmetric nonlinear scaling function with a relatively lower computational complexity can used. However, this arrangement may require a trade-off with other performance metrics.

## 6. Possible challenges and future directions

This section comprehensively discusses the possible challenges in the practical implementation of the proposed control scheme along with potential future research directions.

### 6.1. Possible challenges in practical implementation

Although the practical realization of the LQ-ASMC law was not attempted in this research due to a lack of hardware resources and human subjects. However, implementing the said control procedures on a real system can present a few challenges.

- Real-world sensors are prone to noise. Noisy state measurements can cause erroneous control actions, affecting the overall system performance. Filtering techniques are necessary to handle sensor noise.
- Integrating the controller with existing hardware and software systems can lead to communication delays and unprecedented compatibility problems. Thorough testing and incremental integration approaches can help mitigate these issues.
- The computational tasks associated with the execution of the control procedure are bound to consume significant power. Thermal management is crucial to ensure safe and reliable functionality of the system under all operating conditions.
- Patients exhibit significant variability in insulin sensitivity, glucose metabolism, and response to insulin due to their lifestyle factors (such as stress, exercise, and diet). Hence, identifying personalized physiological models for each patient based on their specific characteristics is a challenging problem. The said variability also makes it quite difficult to develop a one-design-fits-all control strategy.

### 6.2. Future research directions

There is plenty of room for future enhancements in the proposed control scheme.

- In the future, the proposed hybrid control law can be further robustified by introducing power-rate SMC reaching laws in it.
- The switching gain can be adaptively modulated by using adaptive immune feedback.
- Machine learning techniques can be investigated to predict patient’s glucose trends and detect anomalies (or deviations) from expected glucose patterns to adjust the control strategy accordingly.
- Reinforcement learning can be explored to improve the adaptive tuning and performance of the control system.
- In recognition of the value of clinical trials, thorough and ethical experimental validations can be conducted in future phases of this study. In this regard, pilot studies with a small group of patients should be carried out to test the feasibility and initial performance of the controller in a clinical environment.
- The proposed control law proposed can be extended and applied to more comprehensive glucose-insulin dynamic models to investigate the controller’s efficacy and ensure its broader applicability.
- Future research could also attempt to extend this framework to include auxiliary adaptive or predictive elements to supplement the real-time flexibility of the controller design. The exploration of online parameter adaptation of weighting matrices in LQIR is a promising direction in this regard. This arrangement would obviate the necessity of extensive manual tuning.
- Replacing LQIR with the elements of MPC in the proposed framework could also offer better adaptability to external disturbances.
- The controller’s usability in practical scenarios can be further enhanced by including adaptive estimating methods like observers or Kalman filters for the sake of addressing the sensor delays and biased noise.
- Finally, the functionality and convenience of the proposed glucose regulation system can be enhanced by integrating it with wearable technology. For this purpose, the use of non-invasive glucose monitoring technologies, combined with the proposed control algorithm, mechanical insulin delivery, and an interactive user interface in a seamless manner, can be developed to improve patients’ comfort and help them manage their BG levels proactively.

## 7. Conclusion

This article contributes to formulating a robust-optimal regulation technique that employs an LQIR-driven self-adaptive SMC procedure to normalize the BG concentration levels under the influence of meal disruptions and measurement noise. The proposed control procedure beneficially combines the inherent optimal yield of the LQIR with the innate robustness of the SMC. This collaboration is achieved by designing the SMC reaching law using a new sliding surface that depends on the nominal control input of the LQIR. Furthermore, the control law is retrofitted with a simple adaptive framework that dynamically adjusts the switching gain. The self-adjusting gain increases the controller’s adaptability to flexibility modulate the control signal as the BG concentration levels change in the patient’s body. These augmentations significantly improve the controller’s robust tracking against bounded exogenous disturbance while effectively curbing the large IIR demands and upholding the system’s closed-loop stability as the operating conditions change. The LQ-ASMC’s performance assessment is done by conducting customized numerical simulations on the BMM (virtual patient) that examine the system’s BG regulation capacity in the presence of transient meal disturbances, initial hyperglycemic conditions, and measurement noise.

The in-silico analysis of the simulation outcomes validates the enhanced setpoint-tracking ability, time optimality, disturbance compensation capacity, and control input efficiency of the proposed LQ-ASMC law. Despite its robustness and optimal control capabilities, the proposed approach has a few limitations. Firstly, the said control strategy heavily relies on an accurate model of the glucose-insulin system, which can vary significantly between individuals. Secondly, implementing this control law in real-time on hardware systems could face challenges related to computational demands and the need for fast, accurate, and noise-free sensor feedback. However, most of these challenges can be addressed by extending the proposed control scheme as per the research directions proposed earlier in this article.
